# Identification and Characterization of a Leoligin-Inspired Synthetic Lignan as a TGR5 Agonist

**DOI:** 10.1021/acs.jnatprod.5c00059

**Published:** 2025-03-27

**Authors:** Alexander F. Perhal, Patrik F. Schwarz, Thomas Linder, Marko D. Mihovilovic, Michael Schnürch, Verena M. Dirsch

**Affiliations:** † Department of Pharmaceutical Sciences, Division of Pharmacognosy, University of Vienna, Josef-Holaubek-Platz 2, 1090 Vienna, Austria; ‡ Institute of Applied Synthetic Chemistry, TU Wien, Getreidemarkt 9/163, 1060 Vienna, Austria

## Abstract

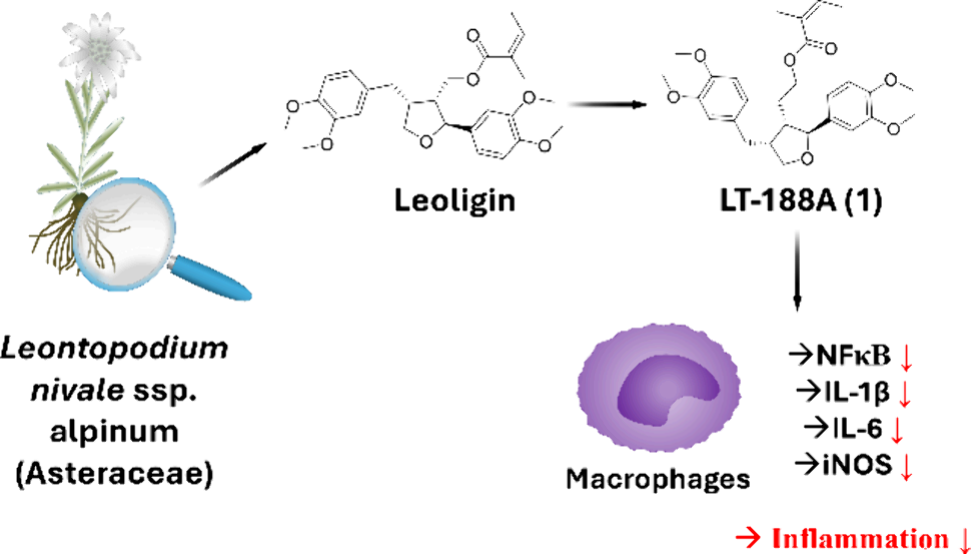

The G-protein coupled bile acid receptor 1 (GPBAR1 or TGR5) is the major cell membrane receptor for bile acids regulating metabolic and immunological functions. Its pharmacological modulation has been shown to alleviate inflammatory diseases, such as type 2 diabetes and atherosclerosis. The naturally occurring lignan leoligin and structural analogues have shown anti-inflammatory effects in vitro. However, the underlying molecular targets are still unknown. In this study, we identify the natural product-inspired synthetic structural analogue of leoligin, LT-188A (**1**), as a novel nonsteroidal TGR5 agonist. LT-188A (**1**) induced cyclic adenosine monophosphate (cAMP) accumulation and cAMP response element (CRE)-dependent luciferase activity in a concentration- and TGR5-dependent manner. Consistently, LT-188A (**1**) inhibited activation of the pro-inflammatory transcription factor nuclear factor κB (NFκB) only in TGR5 expressing cells. In macrophages, LT-188A (**1**) reduced the expression levels of pro-inflammatory cytokines and the production of nitric oxide (NO) as determined by qPCR and the Griess assay, respectively. We showed that LT-188A (**1**) decreased the levels of production of these inflammatory mediators in macrophages. In conclusion, we demonstrate that LT-188A (**1**) is a novel natural product-inspired TGR5 agonist with promising anti-inflammatory in vitro bioactivity in relevant cellular assays representing a promising tool compound with potential for further development.

Metabolic and inflammatory diseases, such as type 2 diabetes or atherosclerosis, are leading causes of morbidity and mortality worldwide with increasing prevalences in a constantly aging society.^1^ Activation of the G-protein coupled bile acid receptor 1 (GPBAR1 or TGR5) was shown to confer profound anti-inflammatory activity,^2,3^ which were proven to prevent ailments, such as atherosclerosis,^4^ type 2 diabetes mellitus^5^ or nonalcoholic steatohepatitis.^6,7^ TGR5 also alleviates liver diseases,^8,9^ increases energy expenditure in metabolic tissue and effectively attenuates diet-induced obesity.^10−12^ Expression in enteroendocrine L cells and pancreatic β-cells mediates the release of the antihyperglycemic peptide hormones glucagon-like peptide 1 (GLP-1) and insulin, respectively, regulating glucose homeostasis.^13−15^

TGR5 belongs to the family of Gα_s_-coupled GPCRs. Its stimulation leads to the generation of the second messenger cyclic adenosine monophosphate (cAMP) with subsequent activation of protein kinase A (PKA), which phosphorylates the transcription factor cAMP response element-binding protein (CREB).^16^ Phosphorylated CREB mediates an increased expression of CRE-regulated target genes, such as the anti-inflammatory IL-10, whereas the expression of pro-inflammatory mediators, such as IL-1β, IL-6 or inducible nitric oxide synthase (iNOS) is reduced.^17^ At the same time, elevated cAMP levels inhibit the activity of inhibitor of nuclear factor kB (IκB) kinases (IKKs), which are activated in response to pro-inflammatory stimuli, such as TNF-α or lipopolysaccharide (LPS). This reduces the phosphorylation of IκB, preventing its degradation and sequestering the transcriptionally inactive nuclear factor κB (NFκB) complex in the cytosol.^18^

The endogenous ligands of the TGR5 receptor are bile acids. TGR5 is the major cell surface receptor for bile acids, next to the nuclear bile acid receptor FXR. The affinities and efficacies of the different endogenous bile acids for the human TGR5 receptor vary significantly, with the microbiome-biotransformed secondary bile acids, i.e., deoxycholic acid (DCA) and lithocholic acid (LCA), showing higher affinities compared to the respective primary bile acids, i.e. cholic acid (CA) and chenodeoxycholic acid (CDCA).^16^ In contrast, CDCA represents the most potent bile acid in activating the nuclear farnesoid X receptor (FXR) while LCA is only weakly active^19^ (Figure 1A). Besides endogenous bile acids, a variety of natural products were identified as TGR5 agonists. The first natural products identified were the pentacyclic triterpenes oleanolic, betulinic, ursolic, and maslinic acid.^20,21^ Other nonsteroidal natural TGR5 agonists include the sesquiterpene coumarins farnesiferol B and microlobidene^22,23^ (Figure 1B). Particularly these nonsteroidal TGR5 ligands represent valuable compounds as they have the potential to act as allosteric modulators beyond the orthosteric receptor binding site.^24^

**Figure 1:**
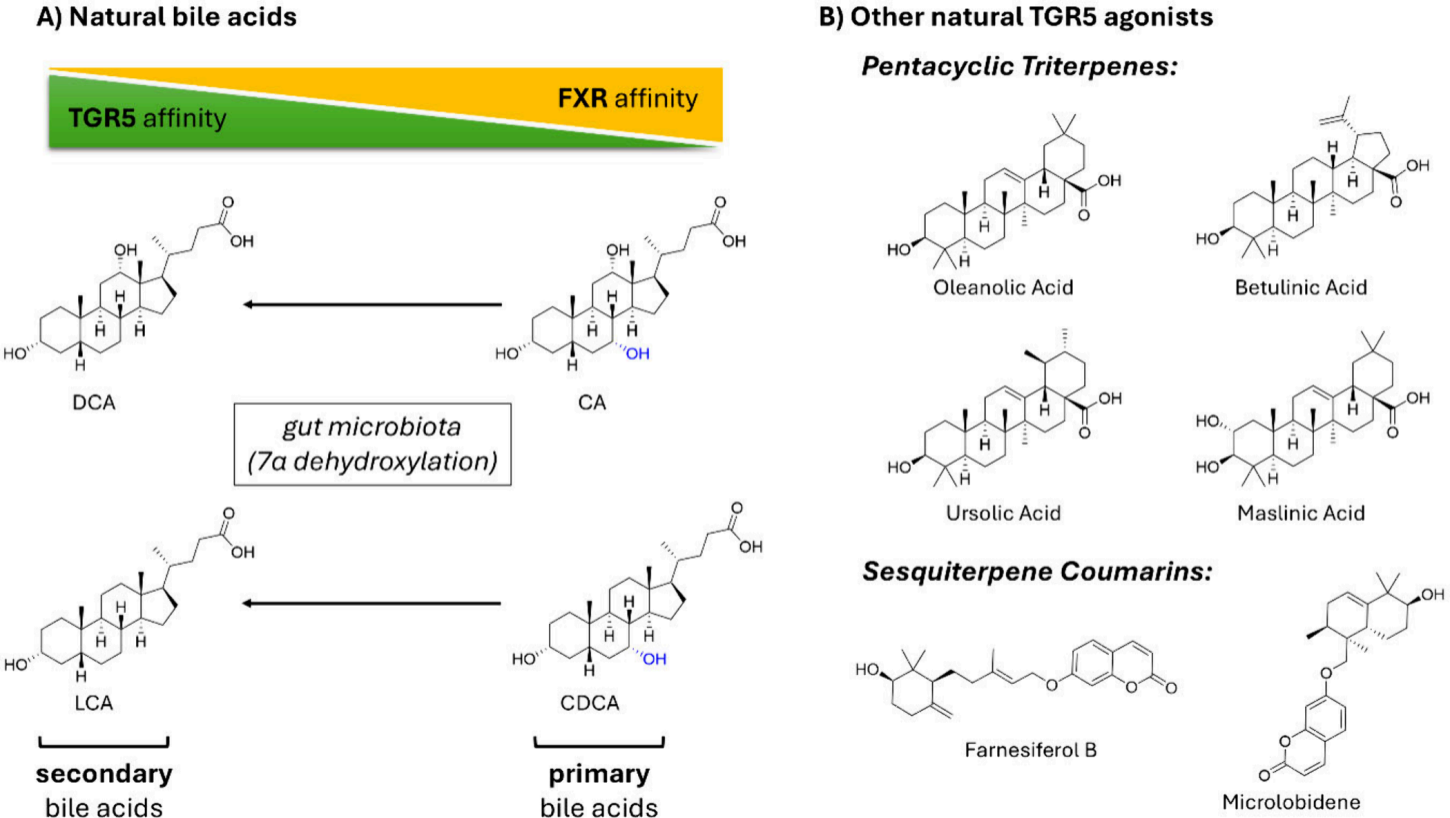
Overview of structures of TGR5 and FXR agonists. (A) Natural bile acids as the endogenous agonists are depicted with reference to their relative affinities for TGR5 and FXR. Primary bile acids, cholic acid (CA) and chenodeoxycholic acid (CDCA), show higher relative affinity for FXR compared to TGR5 and can undergo microbial transformation (i.e., 7α dehydroxylation) by the gut microbiome. The resulting secondary bile acids deoxycholic acid (DCA) and lithocholic acid (LCA) have increased affinities for TGR5 over FXR. The major biotransformation site (7α-OH) is highlighted in blue in the structures of the primary bile acids. (B) Overview of the structures of other previously described natural TGR5 agonists including pentacyclic triterpenes, i.e., oleanolic acid, betulinic acid, ursolic acid, and maslinic acid, as well as the sesquiterpene coumarins farnesiferol B and microlobidene.^19−23^

Leoligin is a naturally occurring tetrahydrofuran-type lignan found in the roots of *Leontopodium nivale* ssp. alpinum (Asteraceae), an alpine plant mainly growing in the European Alps.^25^ Since a stereoselective chemical synthesis has recently been developed,^26,27^ structural analogues of leoligin were synthesized for structure–activity relationship studies. Numerous beneficial pharmacological activities of leoligin and its structural analogues have already been observed, including inhibition of HMG-CoA reductase,^28^ activation of the cholesteryl ester transfer protein (CETP),^29^ and promotion of cholesterol efflux from macrophages.^30^ Notably, it was also found that leoligin and its cognate derivatives can inhibit the transcriptional activity of NFκB in the cells. However, the affected upstream signaling events have not been identified yet.^31^ Furthermore, one structural leoligin analogue has recently been found to transactivate the nuclear bile acid receptor FXR.^32^

These promising bioactivities led us to investigate leoligin structural analogues as potential TGR5 agonists in the present work.

## Results and Discussion

### The Leoligin-Inspired Synthetic Lignan LT-188A (1) Increases Intracellular cAMP Levels in a TGR5-Dependent Manner

**Figure 2:**
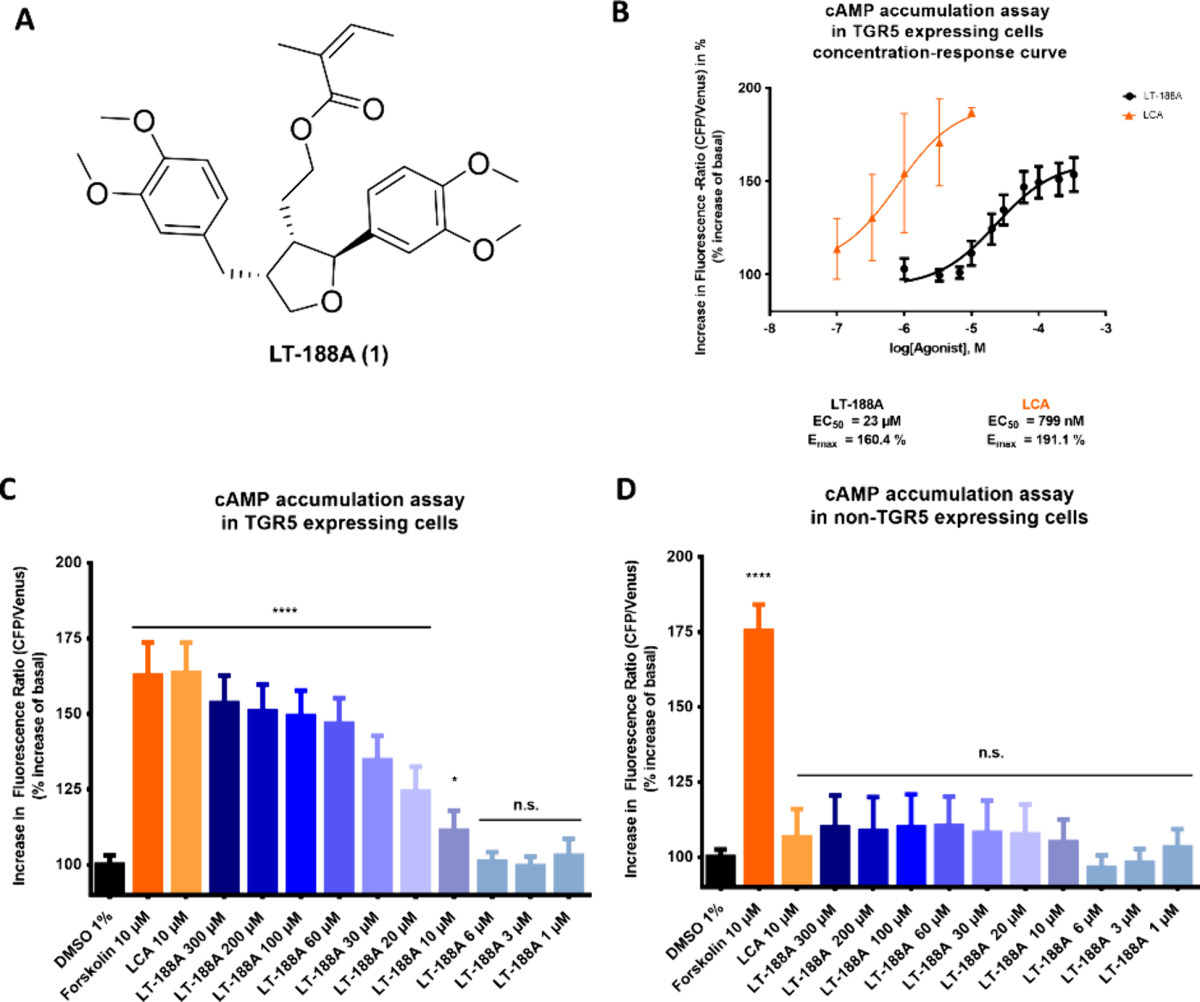
Leoligin derivative LT-188A (**1**) induces intracellular cAMP accumulation in a TGR5-dependent manner. (A) Chemical structure of the C1-homologue of leoligin, LT-188A (**1**). (B, C) Stimulation of TGR5-expressing HEK EPAC cells with LT-188A (**1**) leads to a concentration-dependent increase in the fluorescence ratio of the cAMP-responsive EPAC biosensor in cells. (B) Concentration–response curve of the LT-188A (**1**)-induced effect on cAMP accumulation in TGR5-expressing HEK EPAC cells is shown in black with an EC_50_ of 23 μM and an *E*_max_ of 160.4%. The concentration–response curve for the described TGR5 agonist LCA (EC_50_: 799 nM, *E*_max_: 191.1%) is shown in orange for direct comparison. (D) LT-188A (**1**) does not exert any significant effects on cAMP levels in non-TGR5 expressing HEK EPAC cells in a cAMP accumulation assay. LT-188A (**1**) was tested at the indicated concentrations (300–1 μM) in a cAMP accumulation assay in either TGR5 HEK EPAC cells (C) or non-TGR5 expressing HEK EPAC cells (D). The described TGR5 agonist LCA (10 μM) was included as a positive control. The direct adenylyl cyclase activator Forskolin (10 μM) was included as an additional TGR5-independent positive control. Fluorescence emission ratios (480/526 nm) of cells were measured after 10 min stimulation with compounds in buffer containing 500 μM IBMX and 1 μM roflumilast. Results are expressed as a percentage increase in fluorescence emission ratio compared to vehicle control (DMSO 1%). Bar charts represent the means ± SD of at least three independent biological replicates (*n* ≥ 3) measured in technical triplicates. One-way ANOVA followed by Dunnett’s post hoc test (****p ≤ 0.0001, *p ≤ 0.05, n.s. *p* > 0.05 compared to vehicle control). The concentration–response curve was fitted by using nonlinear regression with a standard Hill coefficient of −1.0.

To identify novel natural product-inspired TGR5 agonists, a screening effort employing an EPAC-based FRET biosensor cAMP accumulation assay in TGR5-expressing cells was conducted with a series of previously generated synthetic leoligin derivatives.^26,27^ This initial screening approach led to the identification of leoligin as a weak TGR5 agonist as well as the C1-homologue of leoligin, LT-188A (**1**), (Figure 2A) as an even more active TGR5 agonist which we then selected for further pharmacological characterization (initial screening results in Figure S3).

To confirm the TGR5 agonistic activity of LT-188A (**1**) and to characterize the concentration dependence of cellular cAMP accumulation, increasing concentrations of LT-188A (**1**) were tested in the cAMP accumulation assay. cAMP levels in TGR5 expressing HEK EPAC cells increased in a concentration-dependent manner in response to LT-188A (**1**) (Figure 2B, C), whereas cAMP accumulation did not significantly increase in non-TGR5 expressing HEK EPAC cells (Figure 2D). Fitting a concentration–response curve for the LT-188A (**1**) response in TGR5 HEK EPAC cells resulted in a determined EC_50_ value of 23 μM and an *E*_max_ value of 160.4% (Figure 2B).

### LT-188A (1) Concentration-Dependently Increases CRE-dependent Transcriptional Activity in a CRE-Luciferase Reporter Gene Assay

**Figure 3:**
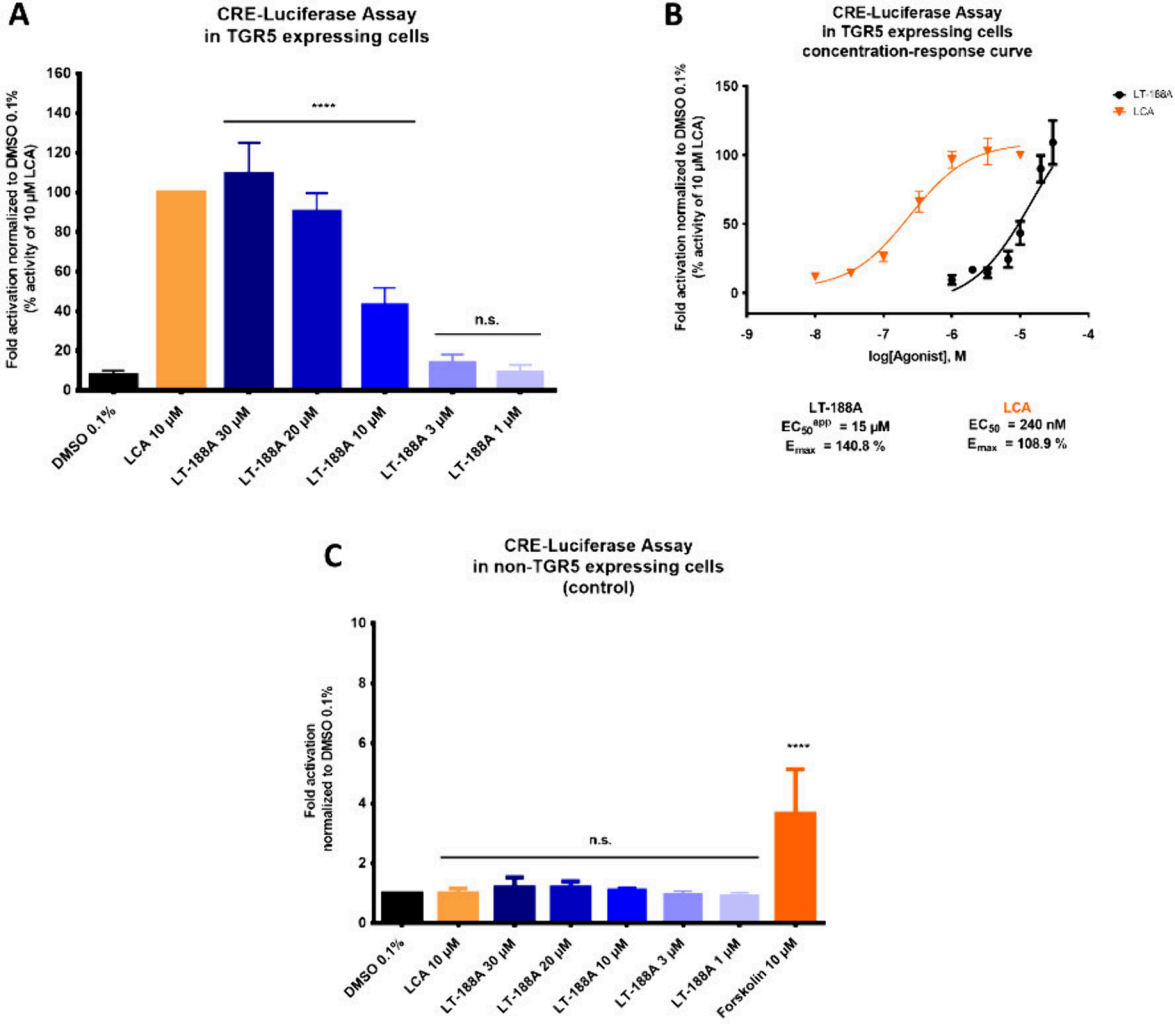
Leoligin derivative LT-188A (**1**) increases luciferase activity in CRE-luciferase reporter gene assays TGR5- and concentration-dependently. (A, B) LT-188A (**1**) leads to a concentration-dependent increase in luminescence levels in TGR5 expressing HEK EPAC cells in CRE-luciferase assays. (B) Concentration–response curve of the LT-188A (**1**)-induced effect on CRE-Luciferase activity in TGR5-expressing HEK EPAC cells is shown in black with an apparent EC_50_ value of 15 μM and an *E*_max_ value of 140.8%. The concentration–response curve for the described TGR5 agonist LCA (EC_50_: 240 nM, *E*_max_: 108.9%) is shown in orange for direct comparison. (C) No effects of LT-188A (**1**) are detectable in non-TGR5 expressing HEK EPAC cells in CRE-luciferase assays. LT-188A (**1**) was tested at the indicated concentrations (30 μM – 1 μM) in CRE-luciferase assays in either TGR5 HEK EPAC cells (A) or non-TGR5 expressing HEK EPAC cells (C). The described TGR5 agonist LCA (10 μM) was included as positive control. Forskolin (10 μM) was included in assays with non-TGR5 expressing HEK EPAC cells as an additional TGR5-independent control. Luminescence signals from the CRE-luciferase reporter were normalized to the EPAC fluorescence levels of cells and vehicle control (DMSO 0.1%) and are expressed as percent activity of the positive control LCA (A) or fold activations (C). Bar charts represent the means ± SD of at least three independent biological replicates (*n* ≥ 3) measured in technical quadruplicates. One-way ANOVA followed by Dunnett’s post hoc test (**** *p* ≤ 0.0001, n.s. *p* > 0.05 compared to vehicle control). The concentration–response curve was fitted using nonlinear regression with a standard Hill coefficient of −1.0. As no upper plateau was yet reached with the highest tested concentration (30 μM) of LT-188A (**1**), a top constraint was set to the highest measured value for fitting the curve and an apparent EC_50_ value (EC_50_^app^) was determined.

Next, a CRE-luciferase reporter gene assay was performed to verify the TGR5 agonistic activity of LT-188A (**1**) downstream of cAMP. LT-188A (**1**) induced a concentration-dependent increase in CRE-dependent luciferase expression in TGR5-expressing HEK EPAC cells (Figure 3A) with an apparent EC_50_ value of 15 μM and an *E*_max_ value of 140.8% (Figure 3B). To confirm the TGR5-dependency of the observed effect, CRE-luciferase assays were performed in non-TGR5 expressing HEK EPAC cells, where no activity of LT-188A (**1**) was detected (Figure 3C).

### LT-188A (1) Does Not Transactivate the Nuclear Farnesoid X Receptor in FXR-Gal4 Luciferase Assays

**Figure 4:**
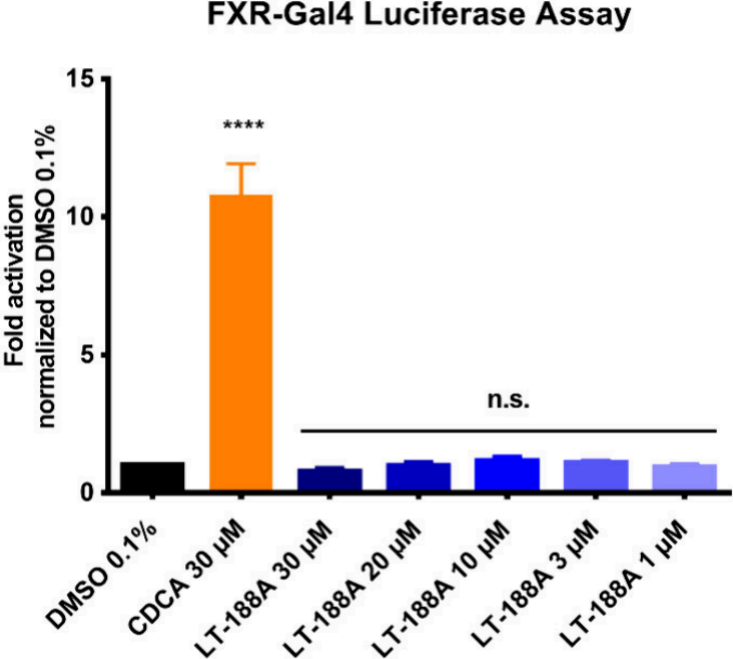
LT-188A (**1**) does not transactivate the nuclear FXR receptor in an FXR-Gal4 luciferase assay. LT-188A (**1**) was tested at the indicated concentrations (30–1 μM) in FXR-Gal4 luciferase assays in HEK293 cells. The potent FXR agonist CDCA (30 μM) was included as a positive control. Luminescence signals from the luciferase reporter were normalized to the eGFP fluorescence levels of cells and vehicle control (DMSO 0.1%) and expressed as fold activation. Bar charts represent the means ± SD of at least three independent biological replicates (*n* ≥ 3) measured in technical quadruplicates. One-way ANOVA followed by Dunnett’s post hoc test (****p ≤ 0.0001, n.s. *p* > 0.05 compared to vehicle control).

To determine a potential effect of LT-188A (**1**) on the nuclear bile acid receptor, FXR, luciferase assays employing a fusion construct of the FXR ligand binding domain with the Gal4 DNA binding domain (FXR-Gal4) in combination with a luciferase reporter under control of upstream activating sequences (UAS) were conducted. Thereby, no significant effects of LT-188A (**1**) on the binding and transactivation of FXR could be observed (Figure 4).

### LT-188A (1) Is Not Cytotoxic in a Resazurin Conversion Assay

**Figure 5:**
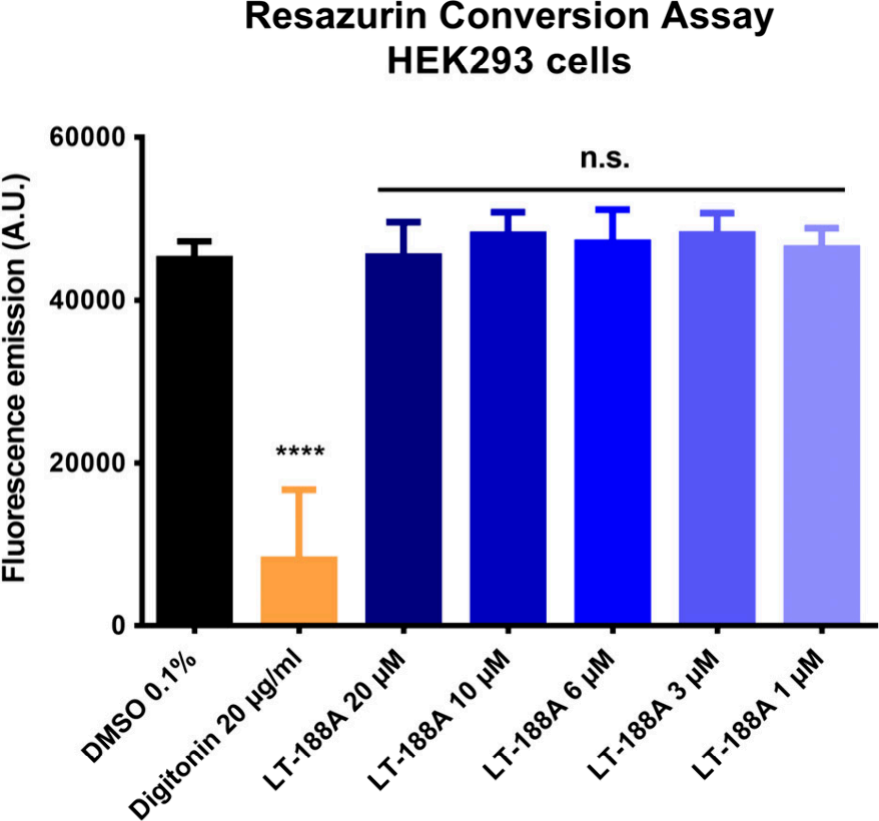
LT-188A (**1**) shows no signs of cytotoxicity up to a concentration of 20 μM in a resazurin conversion assay. To exclude the cytotoxicity of LT-188A (**1**), a resazurin conversion assay was performed. LT-188A (**1**) was tested at the indicated concentrations (20–1 μM) in a resazurin conversion assay in HEK293 cells. Cells were treated with LT-188A (**1**) or the positive control digitonin (20 μg/mL) for 18 h in phenol red-free stripped DMEM before the addition of resazurin (10 μg/mL) for 5 h. Fluorescence emission was measured at an λ_em_ of 590 nm.

To rule out potential cytotoxic effects exerted by LT-188A (**1**) that could bias the results obtained in cellular assays, a resazurin conversion assay was performed. No cytotoxicity of LT-188A (**1**) was observed in HEK293 cells up to a concentration of 20 μM (Figure 5). Therefore, the highest concentration of LT-188A (**1**) used in all functional follow-up experiments, i.e., NFκB-luciferase assays and murine J774A.1 macrophages, was set to 20 μM.

### LT-188A (**1**) Inhibits NFκB Transcriptional Activity in a TGR5-Dependent Manner

**Figure 6:**
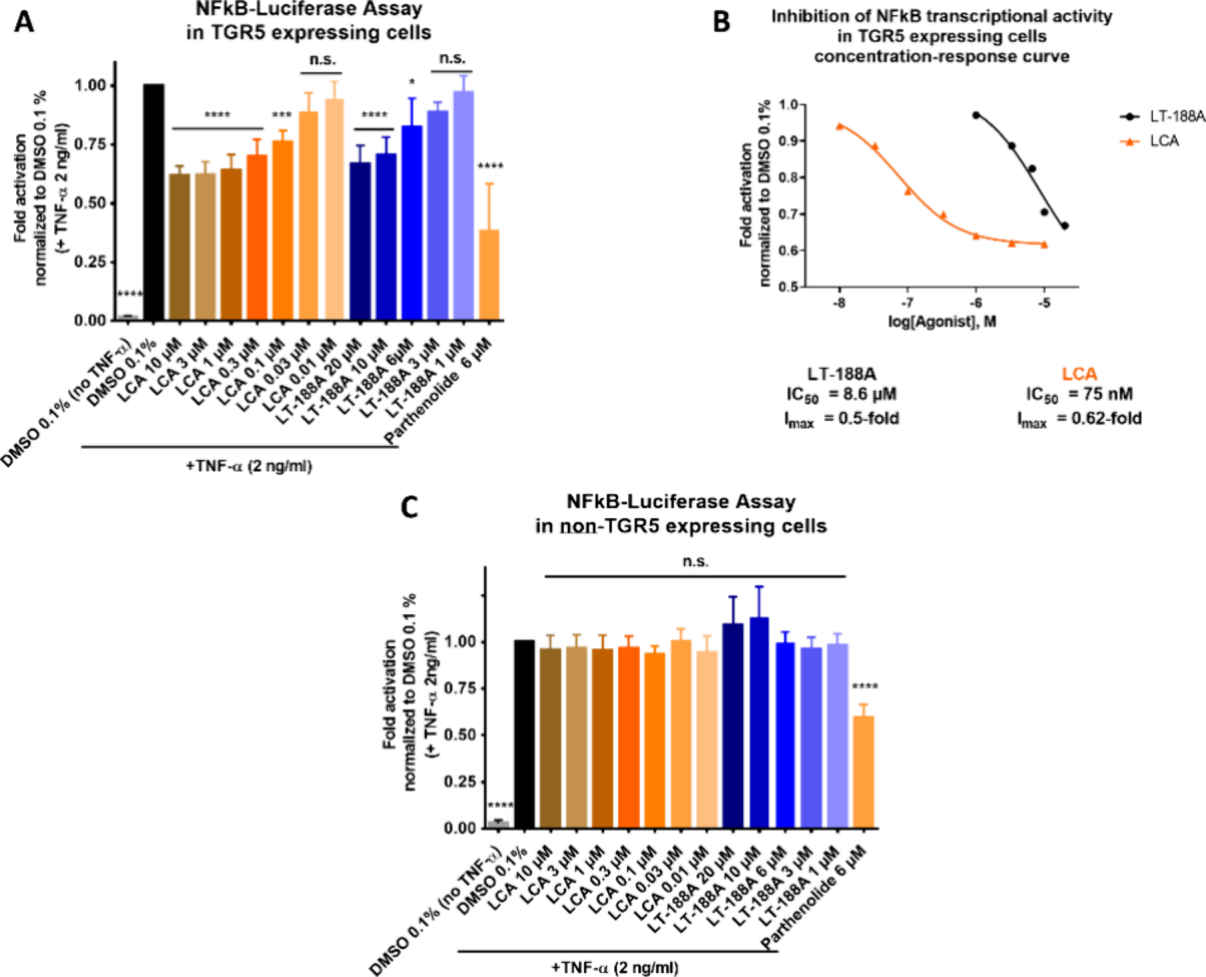
LT-188A (**1**) inhibits NFκB transcriptional activity in a TGR5-dependent manner. (A, B) Treatment with LT-188A (**1**) or the described TGR5 agonist LCA at the indicated concentrations leads to a concentration-dependent inhibition of NFκB transcriptional activity in TGR5 expressing HEK293 cells in NFκB-luciferase assays. (B) Concentration–response curve of the LT-188A (**1**)-induced effect on NFκB-Luciferase activity in TGR5-expressing HEK EPAC cells is shown in black with an IC_50_ of 8.6 μM and an *I*_max_ of 0.5-fold. The concentration–response curve for the described TGR5 agonist LCA (IC_50_: 75 nM, *I*_max_: 0.62-fold) is shown in orange for direct comparison. (C) Treatment with LT-188A (**1**) or the described TGR5 agonist LCA at the indicated concentrations of non-TGR5 expressing wildtype HEK cells does not affect NFκB transcriptional activity in NFκB-luciferase assays. LT-188A (**1**) and LCA were tested at the indicated concentrations in NFκB-luciferase assays in either TGR5-transfected HEK293 cells (A) or non-TGR5 expressing wild-type HEK293 cells (C). The described TGR5 agonist LCA (0.01–10 μM) was included as a positive control. Parthenolide (6 μM), a described NFκB inhibitor, was included in assays as another control. Cells were subsequently stimulated with 2 ng/mL human TNF-α (except for negative control) for 4 h. Luminescence signals from the NFκB-luciferase reporter were normalized to Cell Tracker Green (CTG) fluorescence levels of the cells and vehicle control (DMSO 0.1%) and are expressed as fold activations. Bar charts represent the means ± SD of at least three independent biological replicates (*n* ≥ 3) measured in technical quadruplicates. One-way ANOVA followed by Dunnett’s post hoc test (****p ≤ 0.0001, ***p ≤ 0.001, *p ≤ 0.05, n.s. *p* > 0.05 compared to vehicle control). The concentration–response curve was fitted using nonlinear regression with a standard Hill coefficient of −1.0.

To examine whether TGR5 agonism of LT-188A (**1**) can be linked to an inhibitory activity on the pro-inflammatory transcription factor NFκB, NFκB-luciferase assays were performed, either in TGR5-transfected HEK293 cells or non-TGR5 expressing wild-type HEK293 cells. These experiments revealed that LT-188A (**1**) leads to a concentration-dependent inhibition of NFκB transcriptional activity in TGR5 expressing HEK293 cells with an IC_50_ value of 8.6 μM and an *I*_max_ value of 0.5-fold (Figure 6A, B) while such effects could not be observed in non-TGR5 expressing wildtype HEK293 cells (Figure 6C). As a control for the expression of a functional TGR5 receptor in transfected cells, different concentrations of the described TGR5 agonist LCA were also included in these assays. Likewise, LCA treatment led to a concentration-dependent reduced transcriptional activity of NFκB only in TGR5-expressing cells (Figure 6A) and not in non-TGR5 expressing control (Figure 6C), further highlighting the TGR5-dependent context of the observed effects. Parthenolide as a described inhibitor of NFκB^33^ served as an additional positive control in all NFκB-luciferase assays.

### LT-188A (1) Decreases the Expression of Proinflammatory Cytokines and Nitric Oxide (NO) Production in Murine J774A.1 Macrophages

**Figure 7:**
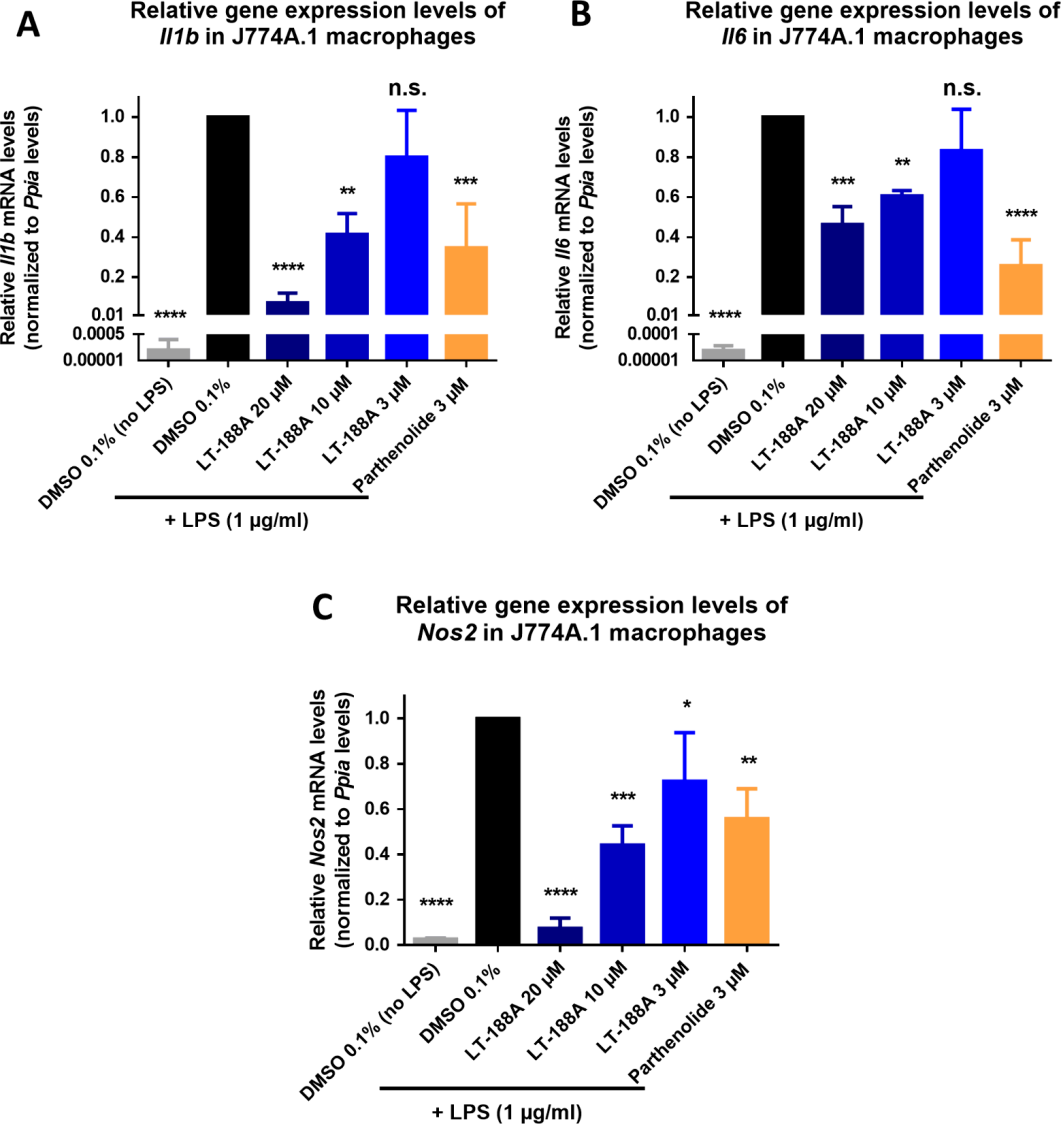
LT-188A (**1**) reduces gene expression levels of the pro-inflammatory mediators *Il1b, Il6* and *Nos2* upon LPS stimulation in J774A.1 macrophages. The mRNA expression levels of pro-inflammatory NFκB target genes *Il1b*, *Il6* and *Nos2* were determined after LT-188A (**1**) treatment and LPS (1 μg/mL) stimulation in J774A.1 macrophages by RT-qPCR. (A) Downregulation of *Il1b* mRNA expression levels after treatment with LT-188A (**1**) at the indicated concentrations (3, 10, and 20 μM) or parthenolide (3 μM). (B) Downregulation of *Il6* mRNA expression levels after treatment with LT-188A (**1**) at the indicated concentrations (3, 10, and 20 μM) or parthenolide (3 μM). (C) Downregulation of *Nos2* expression levels after treatment with LT-188A (**1**) at the indicated concentrations (3, 10, and 20 μM) or parthenolide (3 μM). The expression levels of target genes were normalized to the expression levels of the housekeeping gene *Ppia* and subsequently normalized to the vehicle control (DMSO 0.1%). The described NFκB inhibitor parthenolide (3 μM) was used as a positive control. Bar charts represent expression levels relative to vehicle control (LPS stimulated) expressed as mean ± SD of three biological replicates (*n* = 3) measured in technical triplicates. One-way ANOVA followed by Dunnett’s post hoc test were used for statistical analysis. ****p ≤ 0.0001, ***p ≤ 0.001, **p ≤ 0.01, *p ≤ 0.05, ns *p* > 0.05 compared to vehicle control.

To investigate functional consequences downstream of the TGR5 receptor and NFκB signaling, gene expression levels of important NFκB target genes, such as the pro-inflammatory cytokines *Il-1b*, *Il6* and the inducible NO synthase gene *Nos2* were determined by RT-qPCR. Murine J774A.1 macrophages were used for these experiments because they endogenously express the TGR5 receptor,^4^ whereas human monocyte/macrophage cell lines such as THP-1 cells do not express the TGR5 receptor.^16^ Lipopolysaccharide (LPS) is a potent pro-inflammatory stimulus in J774A.1 macrophages leading to activation of NFκB and was therefore used as stimulus.^34^ J774A.1 macrophages were pretreated with LT-188A (**1**) (20 – 3 μM) or the described NFκB inhibitor parthenolide (3 μM) as positive control for 30 min before stimulation with LPS (1 μg/mL). Gene expression levels were determined by RT-qPCR after 24 h of treatment. Treatment with LT-188A (**1**) resulted in a significant and concentration-dependent decrease in the mRNA expression levels of both pro-inflammatory cytokines *Il1b* (Figure 7A) and *Il6* (Figure 7B), as well as reduced mRNA expression levels of the inducible NO synthase *Nos2* (Figure 7C).

**Figure 8:**
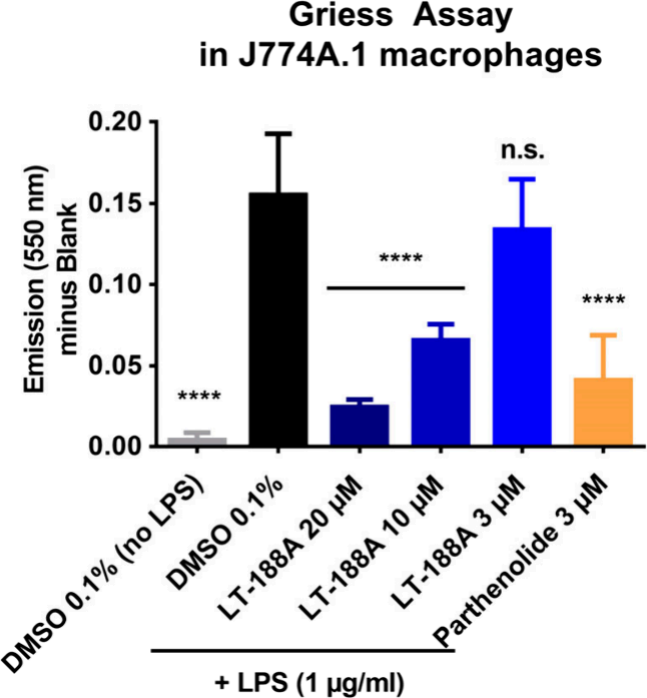
LT-188A (**1**) decreases nitric oxide (NO) production upon LPS stimulation in J774A.1 macrophages. Production of nitric oxide (NO) was quantified in the cell culture supernatants of J774A.1 macrophages upon treatment with LT-188A (**1**) and LPS stimulation by the Griess assay. J774A.1 macrophages were pretreated with LT-188A (**1**) at the indicated concentrations (3, 10, and 20 μM) or the NFκB inhibitor parthenolide (3 μM) for 30 min before stimulation with LPS (1 μg/mL). After 24 h treatment, nitrite levels correlating with the cumulative NO production were determined in cell culture supernatants by the Griess assay where the resulting azo dye was measured at λ_Em_ of 550 nm. Bar charts represent determined nitrite levels expressed as mean ± SD of three biological replicates (*n* = 3) measured in technical triplicate and subtracted background color. One-way ANOVA followed by Dunnett’s post hoc test was used for statistical analysis. ****p ≤ 0.0001, ns *p* > 0.05 compared to vehicle control (DMSO 0.1%, LPS stimulated).

To confirm the functional consequences of the observed reduced mRNA expression levels of *Nos2* in J774A.1 macrophages upon treatment with LT-188A (**1**), nitric oxide (NO) generation after LPS stimulation (1 μg/mL) was quantified by the Griess assay, which quantifies nitrite levels in cell supernatants as a stable marker of released NO. Treatment with LT-188A (**1**) revealed a concentration-dependent inhibition of LPS-stimulated NO production in J774A.1 macrophages (Figure 8) which is in accordance with the observed reduced expression levels of the inducible NO synthase on mRNA level (Figure 7C).

This study identified LT-188A (**1**) as an agonist of the TGR5 receptor. It shows that LT-188A (**1**) increased intracellular cAMP in a TGR5- and concentration-dependent manner with an EC_50_ of 23 μM. LT-188A (**1**) also activated CRE-luciferase activity in a concentration and TGR5-dependent manner with an apparent EC_50_ of 15 μM. NFκB signaling was suppressed with an IC_50_ of 8.6 μM which was also TGR5-dependent. The selectivity of the observed effects for the TGR5 receptor was confirmed in this study by the absence of any significant activities in non-TGR5 expressing cells in cAMP accumulation and luciferase assays. However, future follow-up experiments with TGR5-selective antagonists may give more insight into the pharmacology of LT-188A. LT-188A (**1**) did not bind to the nuclear bile acid receptor FXR.

In CRE-luciferase assays, other signaling pathways, such as the Ca^2+^-calmodulin signaling pathway,^35^ may also ultimately lead to downstream activation of the transcription factor CREB, creating the possibility of biased results. However, the combined results of the cAMP accumulation assay and the CRE-luciferase assay suggest that the activity of LT-188A (**1**) results from TGR5 stimulation, which triggers subsequent downstream signaling events. Although the cAMP accumulation assays provide a direct and quantitative measure of the very proximal event of intracellular cAMP generation following receptor stimulation and the controls performed in non-TGR5 expressing cells, future experiments should ultimately confirm the direct binding of LT-188A (**1**) to the TGR5 receptor.

Bile acids, the endogenous ligands of TGR5, can also activate the nuclear receptor FXR with different affinities.^19^ Importantly, leoligin and another structural analogue have recently been identified to act selectively on FXR, whereas the TGR5 receptor was unaffected.^32^ Therefore, FXR represents a potential obvious off-target of novel TGR5 agonists that is worth testing. One of the primary effects of FXR agonism is the suppression of endogenous bile acid synthesis and consequently a potential limitation of their availability as ligands of the TGR5 receptor.^36^ While selectivity for the TGR5 receptor therefore seems favorable, it has also been shown that dual FXR/TGR5 agonists such as INT-767 can exert synergistic beneficial activity under certain conditions such as diabetic nephropathy.^37^ In this work, we show that LT-188A (**1**) does not bind or induce transactivation of nuclear FXR in an FXR-Gal4 assay. This result is comparable to other natural TGR5 agonists such as triterpenoids which have been shown to be specific for TGR5 over FXR.^20^ Of note, this apparent inactivity of LT-188A (**1**) on FXR in the cellular FXR-Gal4 assay could also be due to a lack of cellular and/or nuclear uptake of the compound.

One of the major underlying signaling pathways modulated by TGR5 stimulation in macrophages is the inhibition of NFκB transcriptional activity.^4^ In line with that, NFκB-luciferase reporter gene assays demonstrated that LT-188A (**1**) inhibited the transcriptional activity of NFκB concentration-dependent and TGR5-dependently. Although these luciferase assays are suitable for detecting inhibitory effects on the transcriptional activity of NFκB, they still require artificial transfection steps and unphysiological expression levels of both the TGR5 receptor and the gene reporter. To study LT-188A (**1**) in a more physiological setting with endogenous expression levels of the TGR5 receptor in macrophages, expression levels of key pro-inflammatory NFκB target genes were determined by RT-qPCR. Since human monocyte/macrophage cell lines such as THP-1 cells do not endogenously express a functional TGR5 receptor,^16^ the murine macrophage cell line J774A.1 was used because it endogenously expresses a functional TGR5 receptor.^4^ Determination of the mRNA expression levels of the proinflammatory cytokines *Il1b* and *IL6,* as well as the mRNA expression levels of the inducible NO synthase *Nos2*, showed that LT-188A (**1**) significantly and concentration-dependently downregulated these LPS-induced mRNAs. Thus, LT-188A (**1**) also prevents the expression of these pro-inflammatory NFκB target genes in cells with endogenous expression levels of the TGR5 receptor. However, as these results are limited to a murine macrophage cell line, follow-up studies should address the effects on target gene expression in human cells.

Finally, to demonstrate that the LT-188A (**1**) treatment of murine J774A.1 macrophages has functional consequences at the protein level, NO production was assessed by the Griess assay. LT-188A (**1**) concentration-dependently decreased NO production upon LPS stimulation in J774A.1 cells which is consistent with the observed reduced mRNA expression levels of *Nos2*.

**Figure 9:**
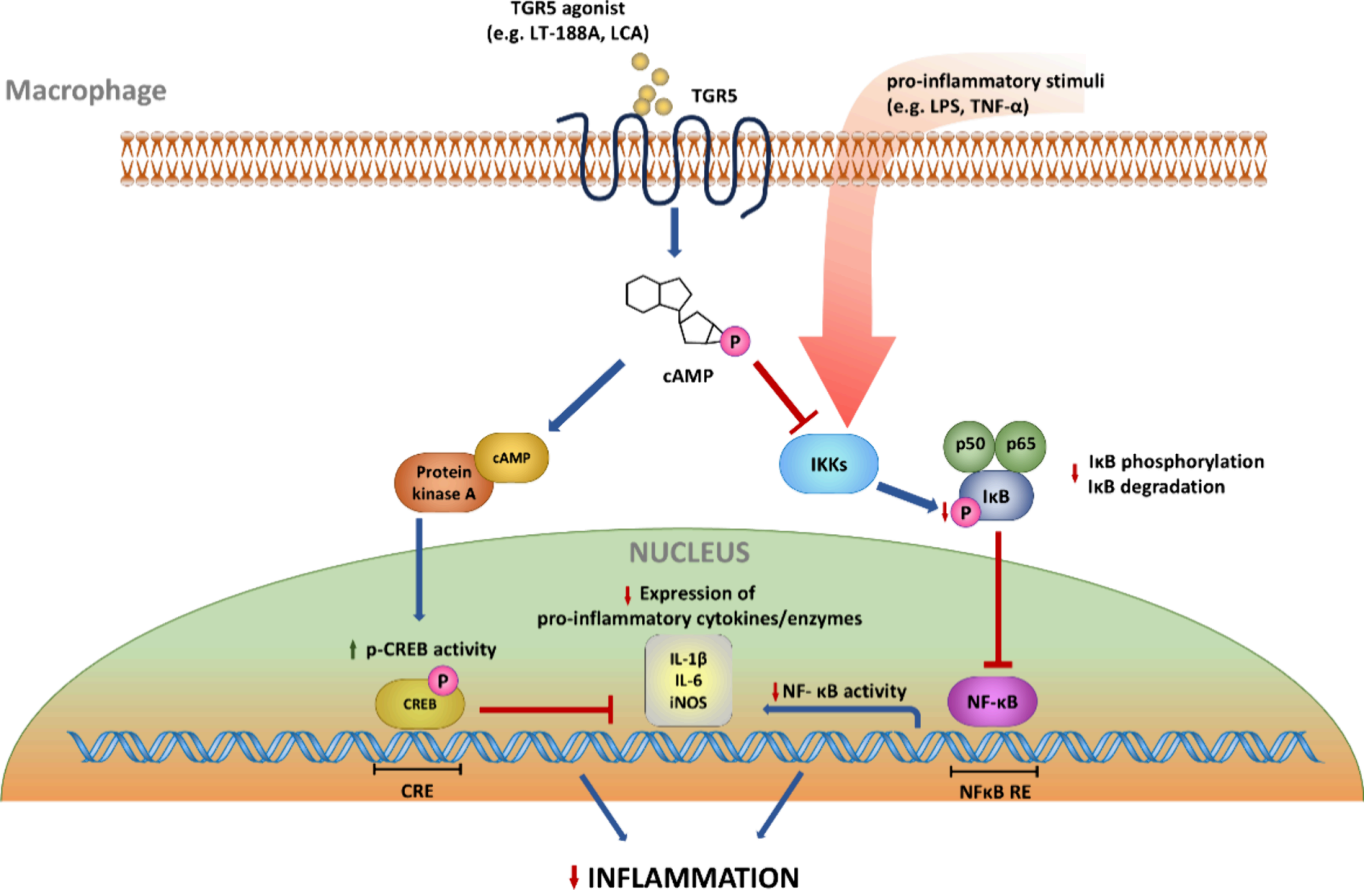
TGR5 agonists and downstream signaling in macrophages mediating anti-inflammatory effects. Activation of the G protein-coupled receptor TGR5 by an agonist, e.g., the bile acid lithocholic acid (LCA) or LT-188A (**1**), on the cell surface of macrophages leads to an intracellular increase in cyclic adenosine monophosphate (cAMP) levels (intermediate steps not depicted). The second messenger cAMP can in turn allosterically activate protein kinase A (PKA) which phosphorylates the transcription factor cAMP response element-binding protein (CREB) leading to increased expression of CRE-regulated target genes, e.g. anti-inflammatory IL-10, and an inhibition of the expression of pro-inflammatory cytokines, such as IL-1β, IL-6, and iNOS. Furthermore, TGR5 downstream signaling inhibits the activity of IκB kinases (IKKs) leading to a decreased phosphorylation state of the inhibitor of nuclear factor kB (IκB) and its stabilization. IκB is subsequently not degraded and keeps the NFκB complex (p50 and p65) sequestered in the cytoplasm preventing the transcription of pro-inflammatory target genes (IL-1β, IL-6, iNOS) in the nucleus. Together both depicted signaling pathways of cAMP downstream of TGR5 receptor activation result in the decreased expression of pro-inflammatory cytokines contributing to the anti-inflammatory effects in macrophages.

In conclusion, this study describes the identification of the semisynthetic compound LT-188A (**1**), obtained by C1-homologization of the natural lignan leoligin, as a novel TGR5 agonist, as demonstrated by its activity in cellular cAMP accumulation and CRE-luciferase assays. Further pharmacological characterization of LT-188A (**1**) showed that this compound is selective for TGR5 over FXR and exerts profound anti-inflammatory activity *in vitro* by inhibiting NFκB transcriptional activity in a TGR5-dependent manner. This reduced NFκB activity results in decreased mRNA expression of the pro-inflammatory target genes *Il1β*, *Il6*, and *Nos2* and reduced iNOS activity in murine macrophages (summarized in Figure 9). LT-188A (**1**) thus represents a promising new natural product-inspired TGR5 agonist available for further semisynthetic optimization. This seems reasonable since the activity of LT-188A (**1**) in cellular assays is still in the micromolar range. Nevertheless, the promising results shown here warrant further studies, e.g., focusing on the promising metabolic effects associated with TGR5 agonism potentially mediated by LT-188A (**1**) or a derivative thereof.

## Experimental Section

### General Experimental Procedures

Human embryonic kidney 293 (HEK293) cells were purchased from the American Type Culture Collection (ATCC, Manassas, VA, USA). Murine J774A.1 macrophages were purchased from LGC PromoChem 1(Teddington, UK). Dulbecco’s modified Eagle medium (DMEM) without phenolred, l-glutamine, and penicillin–streptomycin mixtures were obtained from Lonza (Basel, Switzerland). Fetal bovine serum (FBS) was acquired from biowest (Nuaillé, France). Trypsin, CellTracker Green CMFDA dye, and the High-Capacity cDNA Reverse Transcription Kit were purchased from Thermo Fisher Scientific (Waltham, MA, USA). Enhanced green fluorescent protein (pEGFP-N1) was obtained from Clontech (Mountain View, CA, USA). The CRE-luciferase reporter plasmid pGL4.29[luc2P/CRE/Hygro] was purchased from Promega (Fitchburg, WI, USA). All other plasmids—i.e. FXR-Gal4, pNF-kB-Luc, tk(MH1000)4xLuc and pcDNA3.1-Zeo(+)—were kindly provided by research groups listed in Table S2. TGR5-pcDNA3.1 expression plasmid was generated by subcloning the human TGR5-ORF (NM_170699) into the pcDNA3.1-Zeo(+) vector as described in Table S2. Primers for target gene quantification by RT-qPCR were synthesized by Thermo Fisher Scientific (Waltham, MA, USA), and the sequences are provided in Table S3. The innuPREP RNA Mini Kit 2.0 was obtained from Analytik Jena (Jena, Germany). The Luna Universal qPCR Master Mix was purchased at New England Biolabs (Ipswich, MA, USA). 5X reporter lysis buffer was purchased from Promega (Fitchburg, WI, USA). Adenosine-5′-triphosphate (ATP) disodium salt and ethylenediaminetetraacetic acid (EDTA) were obtained from Carl Roth (Karlsruhe, Germany). D-Luciferin sodium salt was obtained from Synchem (Altenburg, Germany). DMEM supplemented with phenolred, chenodeoxycholic acid (CDCA), coenzyme A (CoA) trilithium salt, Digitonin, dimethyl sulfoxide (DMSO), DL-Dithiothreitol (DTT), lithocholic acid (LCA), LPS from *Escherichia* coli O55:B5, naphthyl-ethylenediamine, phosphoric acid, resazurin sodium salt, roflumilast, and sulfanilamide were purchased from Sigma-Aldrich (St. Louis, MO, USA). Parthenolide was obtained from MedChemExpress (Monmouth Junction, NJ, USA). Human TNF-α was purchased from Miltenyi (Bergisch Gladbach, Germany). Forskolin and IBMX were obtained from Biomol (Hamburg, Germany).Catalog numbers of all commercially obtained materials are summarized in Table S1.

### Cell Lines and Cell Culture

Three different types of HEK293 cells were used in this study: wildtype HEK293, HEK293 cells stably expressing an EPAC-based FRET biosensor for quantification of intracellular cAMP levels (HEK EPAC) as described previously,^38^ as well as HEK EPAC cells stably expressing the TGR5 receptor (TGR5 HEK EPAC). Both wildtype HEK293 and HEK EPAC cells do not endogenously express the TGR5 receptor as judged by their unresponsiveness against known TGR5 agonists (e.g., LCA) in CRE-luciferase and cAMP accumulation assays (Figure S1, C). On the contrary, the stable TGR5 HEK EPAC cell line was generated and screened for its responsiveness to known TGR5 receptor agonists (described in detail in Supporting Information). Cells were cultured in complete DMEM supplemented by 10% FBS, 2 mM l-glutamine, and penicillin–streptomycin (100 U/mL and 100 μg/mL, respectively) at 37 °C and 5% CO_2_. Cells were passaged every 2–3 days and only used up to a maximum in-house passage number of 30 for experiments. HEK293 cells were detached from cell culture flasks using 5 min trypsin/EDTA treatment, while J774A.1 macrophages were detached using a cell scraper. Cell count and viability was regularly checked using an automated cell counter (Vi-Cell XR Cell Viability Analyzer, Beckmann Coulter GmbH, Krefeld, Germany). For some experiments, cells were incubated in 5% charcoal-stripped FBS (stripped media) as stated.

### Synthesis of Leoligin and LT-188A (1)

Leoligin was synthesized according to a literature protocol.^27^ The related compound LT-188A (**1**) differs from leoligin by just one additional CH_2_–group. More specifically, the C8 side chain is extended by one CH_2_–unit. Up to the stage of dimethyllariciresinol (DMLR), the synthesis of LT-188A (**1**) is identical with that of leoligin. First, dimethyllariciresinol was *O*-mesylated quantitatively, affording reaction product LT-169 which readily and reproducibly crystallized upon solvent removal. LT-169 was then converted into nitrile LT-168 by nucleophilic substitution with NaCN in DMSO, which was followed by a two-step reduction, first with DIBAL-H to the intermediate aldehyde and then with NaBH_4_ to the corresponding C1-elongated analogue of dimethyllariciresinol, compound LT-187B. A typical Mitsunobu procedure finally afforded the C1 homologue of leoligin LT-188A (**1**). More detailed information on the synthesis and spectra can be found in the Supporting Information.

### Luciferase Reporter Gene Assay

For luciferase reporter gene assays, HEK293 cells (wildtype, stable HEK EPAC *or* stable TGR5 HEK EPAC) were seeded at a concentration of 8 × 10^6^ cells on 150 mm cell culture dishes 4–5 h prior to transfection. Cells were then transfected with the respective plasmids by calcium phosphate coprecipitation and incubated overnight. For CRE-luciferase assay, 10 μg CRE-luciferase reporter plasmid were transfected into HEK EPAC *or* TGR5 HEK EPAC cells. For the FXR-Gal4 luciferase assay, 5 μg of FXR-Gal4 (FXR ligand binding domain fused to Gal4 DNA binding domain), 5 μg of tk(MH1000)4xLuc (Gal4 UAS luciferase reporter), and 3 μg of pEGFP-N1 (for normalization to cell number and transfection efficacy) were cotransfected into wildtype HEK293 cells. For the NFκB-luciferase assay, wild-type HEK293 cells were either transfected with 5 μg of NFκB-luciferase reporter plasmid alone (non-TGR5-expressing control) or 5 μg of NFκB-luciferase reporter plasmid and 20 μg of TGR5-pcDNA3.1 (TGR5-expressing cells). The next day, the medium was replaced by fresh complete DMEM and cells were allowed to recover from transfection for 4–5 h. For the NFκB-luciferase assay, transfected cells were stained with 2 μM CellTracker Green CMFDA dye (CTG, Thermo Fisher Scientific) within this time frame to stain living cells. Afterward, cells were detached from dishes using trypsin/EDTA and cell suspensions were diluted in stripped DMEM to a concentration of 5 × 10^4^ cells per well onto a 96-well plate where they were treated with vehicle control (0.1% DMSO), respective positive controls or LT-188A (**1**) at the indicated concentrations for 18 h. For the NFκB-luciferase assay, the cells were subsequently stimulated with 2 ng/mL human TNF-α (except for negative control) for another 4 h. After treatment, cells were lysed in 5X reporter lysis buffer (Promega) supplemented with 450 μM coenzyme A and 5 mM dithiothreitol (DTT). Fluorescence emission values (RFU) of eGFP (FXR-Gal4 assay) or CTG (NFκB-luciferase assay) were measured at an emission wavelength of 520 nm (excitation wavelength at 485 nm) using a Tecan Spark spectrophotometer. In the CRE-luciferase assay, the basal fluorescence of the stably transfected EPAC FRET biosensor was used instead of eGFP or CTG staining for cell number normalization and was measured at the same wavelengths. To exclude the possibility that the fluorescence levels of this cAMP-responsive biosensor are still affected after 18 h compound treatment or cell lysis, control experiments in wildtype HEK cells with eGFP transfection or CTG staining were performed, which confirmed the basal fluorescence of the EPAC biosensor as another suitable normalization control in luciferase experiments (data not shown). Luminescence values (RLU) were measured after the addition of ATP and D-Luciferin using a Tecan Spark spectrophotometer. Luminescence values were normalized to the respective fluorescence values to account for differences in cell numbers (RLU/RFU) and subsequently normalized to the vehicle control (0.1% DMSO). Results are expressed as fold activations relative to the vehicle control or as percentage of positive control (10 μM LCA in CRE-luciferase assays).

### Cellular cAMP Accumulation Assay

For investigation of the impact of compound treatments on cellular cAMP levels, HEK293 cells stably transfected with an EPAC-based cAMP sensor (HEK EPAC cells) were employed as described previously.^38^ To determine the selective TGR5-mediated effects of compounds on cellular cAMP levels, a stable HEK EPAC cell line expressing the human TGR5 receptor was generated (described in Supporting Information), while non-TGR5 expressing parent HEK EPAC cells served as a control. HEK EPAC *or* TGR5 HEK EPAC cells were seeded at a concentration of 0.2 × 10^6^ cells per well on a 96-well plate 24 h prior to treatment. Cells were treated with compounds at the indicated concentrations in 1X FURA buffer (138 mM NaCl, 5 mM KCl, 1 mM MgCl_2_, 1.6 mM CaCl_2_, 1 g/L glucose, 20 mM Na-HEPES, pH 7.2) containing 500 μM IBMX (unspecific PDE inhibitor) and 1 μM roflumilast (PDE4-specific inhibitor) for 10 min to allow cAMP accumulation and prevent rapid breakdown within the cells. Thereafter, fluorescence levels of the treated cells were measured using a Tecan Spark photometer (Männedorf, Switzerland) and the FRET ratios of donor/acceptor emission (480 nm/526 nm) were calculated. To account for potential compound autofluorescence, compound dilutions without cells were measured in parallel and subsequently subtracted. FRET ratios were normalized to vehicle control (1% DMSO) and expressed as percent increase in fluorescence ratio compared to vehicle control (100%). As positive controls 10 μM forskolin (direct adenylyl cyclase activator) and 10 μM LCA (TGR5 agonist) were included.

### Evaluation of cytotoxicity by Resazurin Conversion Assay

To evaluate the metabolic activity of cells indicative for potential cytotoxic effects following compound treatment, HEK293 cells were treated with vehicle (0.1% DMSO), digitonin (20 μg/mL) as a cytotoxic positive control, or LT-188A (**1**) at the indicated concentrations in phenol-red-free stripped DMEM for 18 h. The following day, the medium was removed and phenol-red-free stripped DMEM containing 10 μg/mL resazurin sodium salt was added. Cells were incubated for 5 h allowing the enzymatic reduction of resazurin to the fluorescent resorufin, which was eventually measured at an emission wavelength (λ_em_) of 590 nm using a spectrophotometer (Tecan Spark).

### Determination of Gene Expression Levels in J774A.1 Murine Macrophages by RT-qPCR

J774A.1 macrophages were seeded at a concentration of 0.5 × 10^6^ cells per well on a 12-well plate in phenol-red-free complete DMEM. After 24 h incubation, cells were pretreated with vehicle control (0.1% DMSO), positive control (3 μM parthenolide) or LT-188A (**1**) at the indicated concentrations in phenol-red-free stripped DMEM for 30 min. Thereafter, cells were stimulated with LPS (1 μg/mL) or PBS (negative control) and incubated for another 24 h. Total RNA from cells was isolated using the innuPREP RNA Mini Kit 2.0 (Analytik Jena) according to manufacturer’s instructions. The quality and concentration of isolated RNA was checked using a NanoDrop 2000 spectrophotometer (Thermo Fisher Scientific) and stored in aliquots at −70 °C until usage. One μg of RNA was subjected to reverse transcription into cDNA using the High-Capacity cDNA Reverse Transcription Kit (Thermo Fisher Scientific) according to manufacturer’s instructions. RT-qPCR reactions were set up in the Luna Universal qPCR Master Mix (New England Biolabs) using a total of 40 ng of cDNA and 10 μM primer concentration (primer sequences are listed in Table S3) in a final volume of 15 μL. Amplifications were performed in a LightCycler 480 (Roche Diagnostics) consisting of the following reaction steps: initial denaturation (2 min, 95 °C), amplification cycles (denaturation: 15 s at 95 °C, annealing and extension: 1 min at 60 °C). Expression levels of target genes were calculated using the 2^–ΔΔCt^ method^39^ and were normalized to the expression levels of the housekeeping gene *Ppia*, which was previously shown to be a suitable control gene in macrophages.^40^ Expression levels were normalized to the vehicle control (PBS) and expressed as relative fold changes compared to LPS-treated cells.

### Quantification of Nitric Oxide (NO) Production in Murine J774A.1 Macrophages by Griess Assay

To determine the production of nitric oxide (NO) in J774A.1 macrophages, the content of its major stable breakdown product, nitrite was quantified by the Griess Assay.^41^ Briefly, J774A.1 macrophages were seeded at a concentration of 0.5 × 10^6^ cells per well onto a 12 well plate and incubated for 24 h. The next day, cells were preincubated with vehicle (0.1% DMSO), parthenolide (3 μM) or LT-188A (**1**) at the indicated concentrations in phenol-red-free stripped DMEM for 30 min before stimulation with LPS (1 μg/mL), except for the PBS negative control, and an additional incubation period for 24 h. After incubation, 100 μL of the cell culture supernatants were transferred to a 96-well plate and mixed with a freshly prepared 1:1 mixture of the Griess reagents 1% sulfanilamide (in 5% phosphoric acid) and 0.1% N-naphthyl-ethylenediamine. The plate was incubated for 10 min at room temperature before the absorbance was measured at an emission wavelength of 550 nm using a microplate spectrophotometer (Tecan Spark). Samples were measured in technical triplicates per treatment and background (phenol-red-free stripped medium plus Griess reagents) was subtracted.

### Statistical Analysis

All data are presented as mean values ± standard deviation (s.d.) of at least three independent biological replicates (*n* ≥ 3), unless otherwise stated. To determine the statistical significance between treatment groups and controls (vehicle control or positive controls), one-way analysis of variance (ANOVA) followed by a Dunnett’s post hoc test was performed. *p*-values ≤ 0.05 were considered statistically significant. Concentration–response curves were fitted by nonlinear regression with a fixed standard Hill coefficient of −1.0. For concentration–response curves that did not reach an upper plateau with the highest tested concentration, a top constraint was set to highest measured value, and the resulting EC values are expressed as apparent EC_50_ (EC_50_^app^). Effects were considered to be concentration-dependent when the goodness-of-fit value (*R*^2^) of the resulting curve was close to 1.0. All statistical analyses were performed with GraphPad Prism (software version 9.4.1, GraphPad Software Inc.).

## Supporting Information Available

Supplementary data, catalog numbers and providers of commercially obtained materials, list of used plasmids, list of primer sequences used for RT-qPCR, the synthesis of LT-188A (**1**), including ^1^H NMR and ^13^C NMR spectra (PDF)
NMR data (ZIP)
