# Supplementary material for: Identification and Characterization of a Leoligin-Inspired Synthetic Lignan as a TGR5 Agonist
Source: J Nat Prod. 2025 Mar 27;88(4):985–95. doi: 10.1021/acs.jnatprod.5c00059 (PMC12038849; doi:10.1021/acs.jnatprod.5c00059)
Supplement: Supplementary file 1 — np5c00059_si_001.pdf [file np5c00059_si_001.pdf]

Supporting Information

Identification and Characterization of a Leoligin-inspired Synthetic Lignan as a TGR5 Agonist

AUTHOR NAMES

Alexander F. Perhal[1], Patrik F. Schwarz[1], Thomas Linder[2], Marko D. Mihovilovic[2], Michael Schnürch[2], Verena M. Dirsch[1]*

AUTHOR ADDRESS

[1]Department of Pharmaceutical Sciences, Division of Pharmacognosy, University of Vienna, Josef-Holaubek-Platz 2, 1090 Vienna, Austria

[2]Institute of Applied Synthetic Chemistry, TU Wien, Getreidemarkt 9/163, 1060 Vienna, Austria

[*]Correspondence: Alexander F. Perhal (alexander.perhal@univie.ac.at)

Contents of SI:

- SUPPLEMENTARY DATA (page 2)
- Catalog numbers and providers of commercially obtained materials. (page 6)
- Donated and self-subcloned plasmids with providers or subcloning strategies. (page 9)
- Primer used for RT-qPCR. (page 10)
- Supplementary information on the synthesis of LT-188A (1) (page 11)
- ^{1}H-NMR spectra of LT-188A (**1**). (page 21)
- ^{13}C-NMR spectra of LT-188A (**1**). (page 22)
- Supplementary References (page 22)

SUPPLEMENTARY DATA

Generation and pharmacological characterization of a stable TGR5 HEK EPAC cell line

HEK EPAC cells do not endogenously express a functional TGR5 receptor given by their unresponsiveness against known TGR5 agonists (e.g. LCA). Therefore, in order to generate a stable HEK EPAC cell line expressing the TGR5 receptor, human TGR5 expression plasmid (hTGR5-pcDNA3.1-Zeo(+), Table S2) was first linearized by restriction digestion using PciI (New England Biolabs). Linearized plasmid DNA was purified from agarose gel using the Monarch® DNA Gel Extraction Kit (New England Biolabs) and was transfected into HEK EPAC cells using calcium phosphate co-precipitation. The following day, transfected cells were then subjected to selection by zeocin (400 µg/mL, Thermo Fisher Scientific) until all untransfected were killed and resistant cells began to grow again. By then cells were transferred to 96-well plates at a concentration of 1 cell per well to achieve monoclonal cell lines. Once single cell colonies almost reached confluency, the cell clones were screened for their responsiveness against the TGR5 agonist LCA in the cAMP accumulation assay employing the EPAC-based FRET biosensor (described under Materials and Methods in the manuscript). Identified positive clones were subsequently frozen in complete DMEM supplemented by 10% DMSO in liquid nitrogen for later usage. In this study TGR5 HEK EPAC **clone 1** was used exclusively and therefore further pharmacologically characterized both with CRE-Luciferase Assay (**Figure S1**) and cAMP accumulation assay (**Figure S2**).

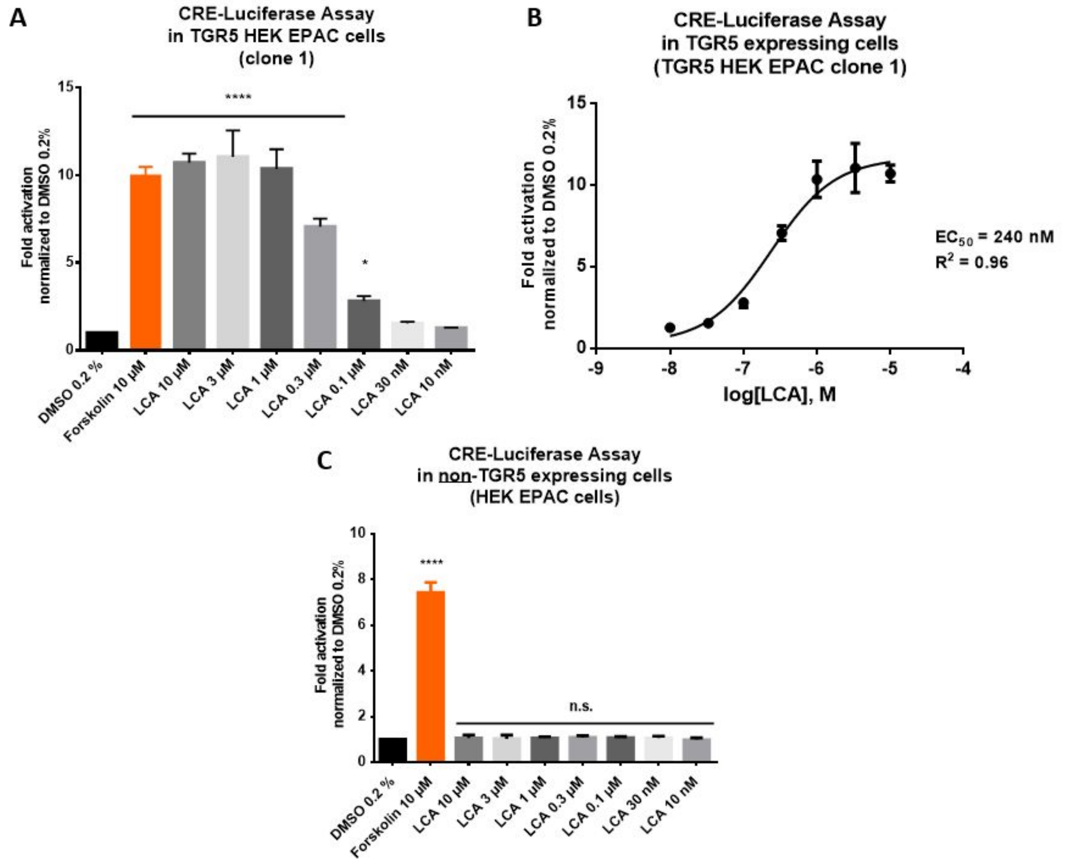

Figure S1. Pharmacological characterization of the stable TGR5-expressing HEK EPAC clone 1 (**A, B**) or non-TGR5 expressing parent HEK EPAC cells (**C**) by CRE-Luciferase Assay. CRE-Luciferase-transfected TGR5 HEK EPAC or HEK EPAC cells were treated with the indicated concentrations of the TGR5 agonist LCA for 18 hours. The luminescence values from CRE-Luciferase reporter were normalized to fluorescence levels of cellular EPAC sensor and expressed as fold activation normalized to vehicle control (0.2% DMSO). The direct adenylate cyclase activator Forskolin (10 µM) was included as a TGR5-independent control. (**A**) Treatment of TGR5 HEK EPAC cells (clone 1) lead to a concentration-dependent increase in luminescence signals with increasing concentration of the TGR5 agonist LCA with a determined EC_{50} value of 240 nM for LCA which is comparable to published values[1] (**B**). (**C**) Non-TGR5 expressing HEK EPAC cells showed no response to LCA treatment at the same concentrations confirming the absence of functional TGR5 receptor in this cell line.

Data are presented as means ± SD of three biological replicates (n = 3) measured in technical quadruplicates. One-way ANOVA followed by Dunnett's post hoc test were used for statistical analysis. **** $p \leq 0.0001$, * $p \leq 0.05$, ns $p > 0.05$ compared to vehicle control. The concentration-response curve was fitted by nonlinear regression using a standard Hill coefficient of −1.0.

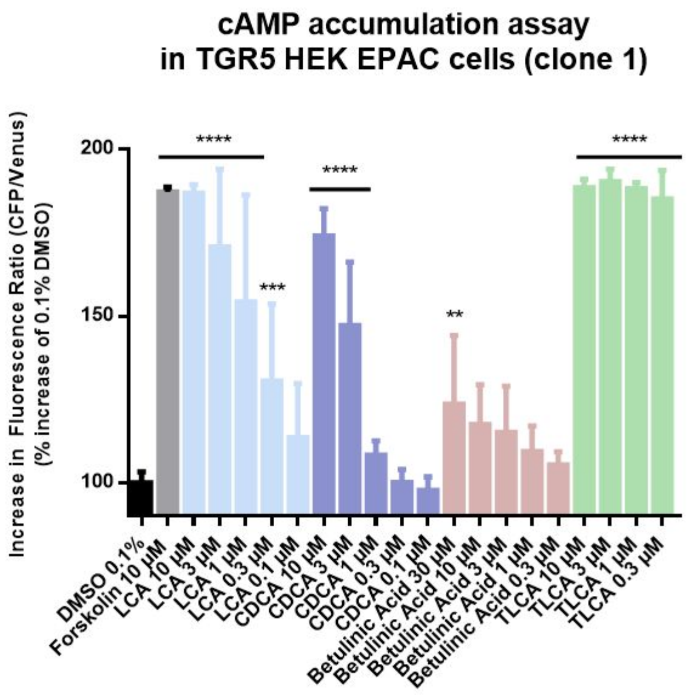

Figure S2. Pharmacological characterization of the stable TGR5-expressing HEK EPAC clone 1 by the cAMP accumulation assay. Treatment of TGR5 HEK EPAC cells with different established TGR5 agonists (bile acids LCA, CDCA, and TLCA, as well as the natural triterpene betulinic acid[2]) lead to concentration-dependent accumulation of cAMP levels in cells. Cells were treated with compounds in the indicated concentrations in 1X FURA buffer containing the PDE inhibitors 500 µM IBMX and 1 µM roflumilast for 10 minutes. Afterwards, fluorescence levels of the cells were measured and FRET ratios calculated (480 nm/526 nm) and normalized to vehicle control (0.1% DMSO). Results are

expressed as percent increase in fluorescence ratio compared to vehicle control (100%). Data are presented as means ± SD of three biological replicates (n = 3) measured in technical triplicates. One-way ANOVA followed by Dunnett's post hoc test were used for statistical analysis. **** $p \leq 0.0001$, *** $p \leq 0.001$, ** $p \leq 0.01$ compared to vehicle control.

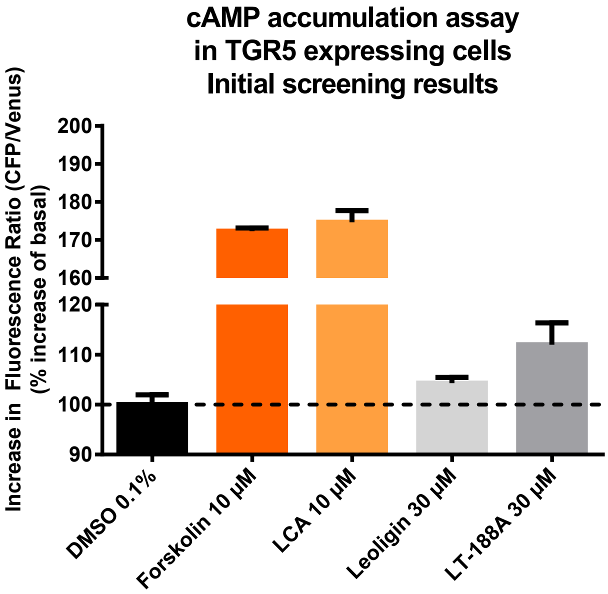

Figure S3. Initial screening in a cAMP accumulation assay identified leoligin as a weak TGR5 agonist and LT-188A (**1**) as a more active analogue thereof. Initial screening result (n=1) of leoligin and LT-188A (**1**) in cAMP accumulation assays revealed leoligin as a weak TGR5 agonist (104.3 ± 1.13 %) and LT-188A (**1**) as a more active analogue thereof (112 ± 4.36 %). Leoligin or LT-188A (**1**) were tested at the indicated concentrations (30 µM) in cAMP accumulation assays in TGR5 HEK EPAC cells. The described TGR5 agonist LCA (10 µM) was included as positive control. Forskolin (10 µM) was included as an additional TGR5-independent control.

Table S1. Catalog numbers and providers of commercially obtained materials.

**Material**	**Catalog number**	**Provider**
1-Methyl-3-Isobutylxanthine, Isobutylmethylxanthine (IBMX)	Cay13347	Biomol
5X reporter lysis buffer	E3971	Promega
Adenosine-5'-triphosphate (ATP) disodium salt	HN35.2	Carl Roth
Betulinic Acid	B8936	Sigma-Aldrich
CellTracker® Green CMFDA dye	C7025	Thermo Fisher Scientific
Chenodeoxycholic acid (CDCA)	C9377	Sigma-Aldrich
Coenzyme A (CoA) trilithium salt	C3019	Sigma-Aldrich
Digitonin	D141	Sigma-Aldrich
Dimethyl sulfoxide (DMSO)	M81802	Sigma-Aldrich
DL-Dithiothreitol (DTT)	43815	Sigma-Aldrich
D-Luciferin sodium salt	BC218	Synchem
DMEM	D6546	Sigma-Aldrich
DMEM phenolred-free	12-917F<br>BE12-917F	Lonza
ethylenediaminetetraacetic acid (EDTA)	8043.2	Carl Roth

FBS	S1810 (batch number: S00CN)	biowest
Forskolin	Cay11018-5	Biomol
HEK293 cells	CRL-1573	ATCC
High-capacity cDNA Reverse Transcription Kit	4368814	Thermo Fisher Scientific
human TNF-α	130-094-014	Miltenyi Biotec
innuPREP RNA Mini Kit 2.0	845-KS-2040250	Analytik Jena
J774A.1 cells	TIB-67	LGC PromoChem / ATCC
L-glutamine	BE17-605E	Lonza
Lithocholic acid (LCA)	L6250	Sigma-Aldrich
LPS from *Escherichia* coli O55:B5	L2880	Sigma-Aldrich
Luna Universal qPCR Master Mix	M3003E	New England Biolabs
N-Naphthyl-ethylenediamine	8.06206	Sigma-Aldrich
Parthenolide	HY-N0141	MedChemExpress
PciI	R0655S	New England Biolabs
pEGFP-N1	6085-1	Clontech
Penicillin-Streptomycin mixture	DE17-602E	Lonza
pGL4.29[luc2P/CRE/Hygro]	E8471	Promega
Phosphoric acid	79617	Sigma-Aldrich

Resazurin sodium salt	199303	Sigma-Aldrich
Roflumilast	SML1099	Sigma-Aldrich
Sulfanilamide	S9251	Sigma-Aldrich
Taurolithocholic acid (TLCA) sodium salt	T6260	Sigma-Aldrich
Trypsin	27250-018	Thermo Fisher Scientific
Zeocin™ Selection Reagent	R25001	Thermo Fisher Scientific

Table S2. Donated and self-subcloned plasmids with providers or subcloning strategies.

Plasmid	Provider / Cloning Strategy
FXR-Gal4	Prof. Daniel Merk (Department of Pharmacy, Ludwig Maximilians University Munich, Munich, Bavaria, Germany)
pNF-kB-Luc	Prof. Angelika Vollmar (Department of Pharmacy, Ludwig Maximilians University Munich, Munich, Bavaria, Germany)
tk(MH1000)4xLuc	Prof. Ronald Evans (Salk Institute for Biological Studies, La Jolla, California, USA)
hTGR5-pcDNA3.1-Zeo(+)	GPBAR1 transcript variant 3 (NM_170699) Human Untagged cDNA Clone (SC123312) was obtained from OriGene Technologies GmbH (Herford, Germany) and subcloned into the pcDNA3.1 Zeo(+) expression vector using the restriction sites NotI/NotI. Successful subcloning was confirmed by Sanger sequencing.

Table S3. Primer used for RT-qPCR.

**Primer name**	**Sequence (5'-3')**	**Provider**
Ppia fwd.	CCAAGACTGAATGGCTGGATG	Thermo Fisher Scientific
Ppia rev.	TGTCCACAGTCGGAAATGGTG	Thermo Fisher Scientific
Nos2 fwd.	CAGAGGACCCAGAGACAAGC	Thermo Fisher Scientific
Nos2 rev.	TGCTGAAACATTTCCTGTGC	Thermo Fisher Scientific
Il1b fwd.	CAACCAACAAGTGATATTCTCCATG	Thermo Fisher Scientific
Il1b rev.	GATCCACACTCTCCAGCTGCA	Thermo Fisher Scientific
Il6 fwd.	GAGGATACCACTCCCAACAGACC	Thermo Fisher Scientific
Il6 rev.	AAGTGCATCATCGTTGTTCATACA	Thermo Fisher Scientific

Supplementary information on the synthesis of LT-188A (1)

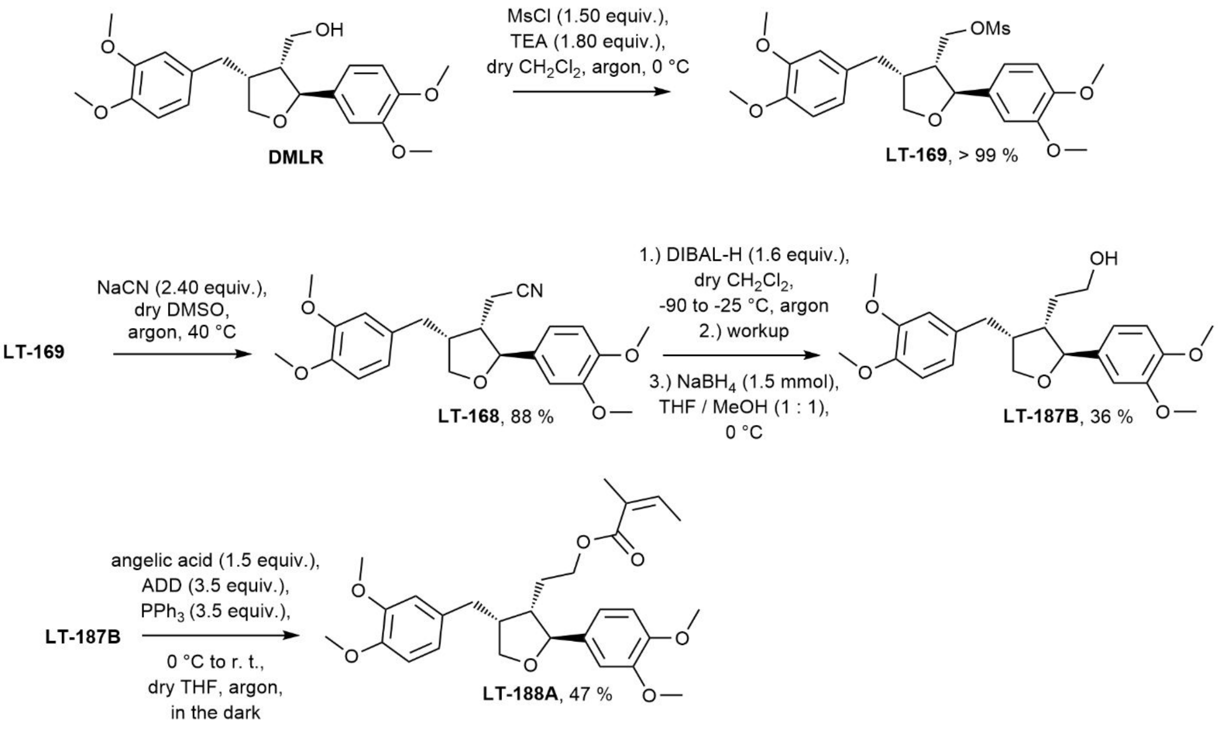

Figure S4. Synthesis of the leoligin analogue LT-188A (**1**). Synthetic route to the leoligin analogue LT-188A (**1**) starting from dimethyllariciresinol (DMLR). First, dimethyllariciresinol was O-mesylated quantitatively, affording reaction product LT-169 which readily and reproducibly crystallized upon solvent removal. LT-169 was then converted into nitrile LT-168 by nucleophilic substitution with NaCN in DMSO, which was followed by two-step reduction, first with DIBAL-H to the intermediate aldehyde and then with $NaBH_4$ to the corresponding C1-elongated analogue of dimethyllariciresinol, compound LT-187B A typical Mitsunobu procedure finally afforded the C1 homolog of leoligin LT-188A (**1**).

((2*S*,3*R*,4*R*)-4-(3,4-Dimethoxybenzyl)-2-(3,4-dimethoxyphenyl)tetrahydrofuran-3-yl)methyl methanesulfonate (**LT-169**)

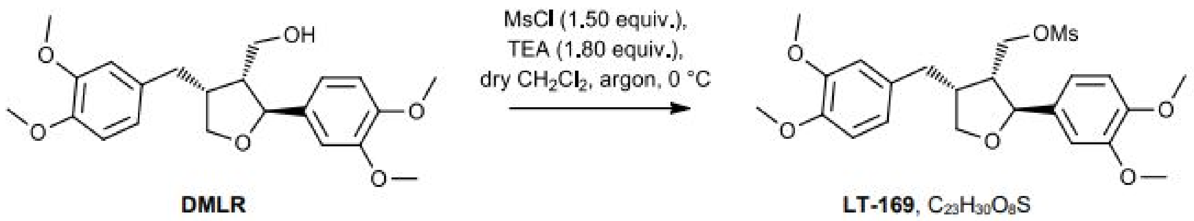

Figure S5. O-mesylation of dimethyllariciresinol (DMLR) to the intermediate LT-169.

***Procedure**:* a reaction vessel was charged with a stirring bar, starting material **DMLR** (108.4 mg, 0.279 mmol, 1.00 equiv.) and evacuated and back-filled with argon using standard Schlenk technique. Dry CH_2Cl_2 (1 mL) and triethylamine (70 µL, 0.502 mmol, 1.80 equiv.) were then added *via* syringe and the mixture was cooled to 0 °C in an ice bath. This was followed by the addition of mesyl chloride (32 µL, 0.419 mmol, 1.50 equiv.) *via* syringe and the reaction then stirred for 2 h while allowed to warm to room temperature. Aqueous HCl (0.5 M, 10 mL) was added dropwise, followed by extraction with Et_2O (4 x 20 mL) and EtOAc (2 x 20 mL). The combined organic phases were treated with water (10 mL), brine (10 mL), dried with Na_2SO_4, filtered and the solvent was evaporated. To the residue was again added Et_2O (10 mL) and then sonicated for approximately 3 min, which caused the reaction product to crystallize. Evaporation at room temperature then afforded the title compound **LT-169**.

Yield: 130.1 mg, > 99 %

Appearance: slightly off-white crystals

Melting range: 114.0 – 116.0 °C

***R*$_f$ (silica)**: 0.62 (EtOAc)

$[\alpha]_D^{25}$: +19.9 (c 0.64, MeOH / $CHCl_3$, 5 : 1)

LC-HRMS (ESI): calculated for M+Na^+: 489.1559, found: 489.1568, Δ: 1.82 ppm

(log *P*)$_{calc}$: 2.77 ± 0.51

^{1}H NMR (200 MHz, $CDCl_3$): δ 2.49 – 2.97 (m, 4H, H3, H4, C4-CH_2), 2.99 (s, 3H, OSO_2CH_3), 3.75 (dd, ^2J = 8.7 Hz, ^3J = 6.4 Hz, 1H, H5), 3.86 (s, 3H, Ar-OCH_3), 3.87 (s, 6H, Ar-OCH_3), 3.89 (s, 3H, Ar-OCH_3), 4.09 (dd, ^2J = 8.7 Hz, ^3J = 6.4 Hz, 1H, H5), 4.32 (dd, ^2J = 9.9 Hz, ^3J = 6.9 Hz, 1H, C3-CH), 4.51 (dd, ^2J = 10.0 Hz, ^3J = 6.8 Hz, 1H, C3-CH), 4.84 (d, ^3J = 6.1 Hz, 1H, H2), 6.67 – 6.91 (m, 6H, Ar-H).

^{13}C NMR (50 MHz, $CDCl_3$): δ 33.2 (t, C4-<u>C</u>), 37.6 (q, OSO_2CH_3), 42.3 (d, C4), 49.6 (d, C3), 56.1 (q, 4 x Ar-OCH_3), 67.7 (t, C3-<u>C</u>), 72.8 (t, C5), 82.6 (d, C2), 109.0 (d, C2'), 111.2 (d, C5'), 111.5 (d, C5''*), 112.0 (d, C2''*), 118.2 (d, C6'), 120.6 (d, C6''), 132.2 (s, C1''), 134.3 (s, C1'), 147.8 (s, C4''), 148.8 (s, C4'), 149.2 (s, C3''), 149.3 (s, C3').

2-((2*S*,3*S*,4*R*)-4-(3,4-Dimethoxybenzyl)-2-(3,4-dimethoxyphenyl)tetrahydrofuran-3-yl)acetonitrile (**LT-168**):

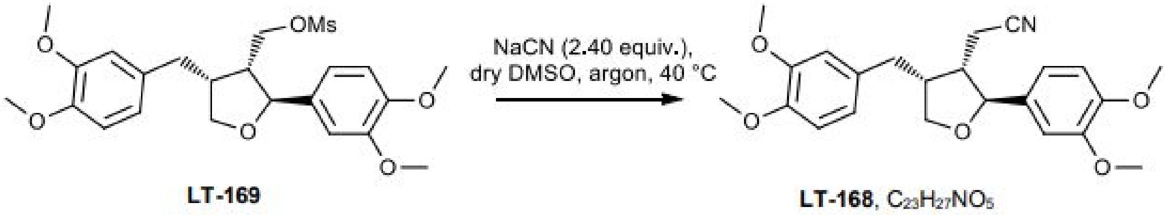

Figure S6. Conversion of LT-169 into the nitrile LT-168 by nucleophilic substitution.

Procedure: a reaction vessel was charged with a stirring bar, starting material **LT-169** (95.9 mg, 0.206 mmol, 1.00 equiv.), dried NaCN (14.1 mg, 0.288 mmol, 1.40 equiv.) and evacuated and back-filled with argon using standard Schlenk technique. Then, dry DMSO (1.5 mL) was added *via* syringe, the mixture was stirred at 40 °C for 27 h and then allowed to remain at room temperature overnight. Water (5 mL) was added, followed by Et_2O (25 mL), the layers were separated and the aqueous phase was re-extracted with Et_2O (3 x 15 mL). The combined organic phases were treated with brine (3 mL), dried with Na_2SO_4, filtered and the solvent was evaporated. Purification by flash column chromatography (18 g silica, flow rate 20 mL / min, EtOAc / LP, 10 : 90 to 60 : 40 in 60 min) afforded the title compound **LT-168**.

**Yield**:	71.6 mg, 88 %
**Appearance**:	colorless oil
***R*$_f$ (silica)**:	0.53 (EtOAc)
**[α]$_D^{23}$**:	+28.4 (c 0.67, MeOH)
**LC-HRMS (APCI)**:	calculated for M+Na$^+$: 420.1781, found: 420.1789, Δ: 1.90 ppm

(log *P*)$_{calc}$: 3.06 ± 0.44

^{1}H NMR (200 MHz, CDCl$_3$): δ 2.66 – 2.42 (m, 4H, H3, C3-CH$_2$, C4-CH), 2.97 – 2.71 (m, 2H, H4, C4-CH), 3.79 (dd, ^2J = 8.9 Hz, ^3J = 5.6 Hz, 1H, H5), 3.87 (s, 3H, Ar-OCH$_3$), 3.88 (s, 3H, Ar-OCH$_3$), 3.88 (s, 3H, Ar-OCH$_3$), 3.90 (s, 3H, Ar-OCH$_3$), 4.12 (dd, ^2J = 8.9 Hz, ^3J = 6.6 Hz, 1H, H5), 4.70 – 4.76 (m, 1H, H2), 6.89 – 6.68 (m, 6H, Ar-H).

^{1}H NMR (50 MHz, CDCl$_3$): δ 16.3 (t, C3-C), 33.2 (t, C4-C), 42.5 (d, C4), 47.3 (d, C3), 56.1 (q, 4 x Ar-OCH$_3$), 72.3 (t, C5), 84.6 (d, C2), 108.8 (d, C2'), 111.2 (d, C5'), 111.5 (d, C5''*), 112.0 (d, C2''*), 118.3 (d, C6'), 118.8 (s, C3-CH$_2$-CN), 120.7 (d, C6''), 131.6 (s, C1''), 133.3 (s, C1'), 147.9 (s, C4''), 149.1 (s, C4'), 149.2 (s, C3''), 149.5 (s, C3').

2-((2*S*,3*S*,4*R*)-4-(3,4-Dimethoxybenzyl)-2-(3,4-dimethoxyphenyl)tetrahydrofuran-3-yl)ethanol (**LT-187B**):

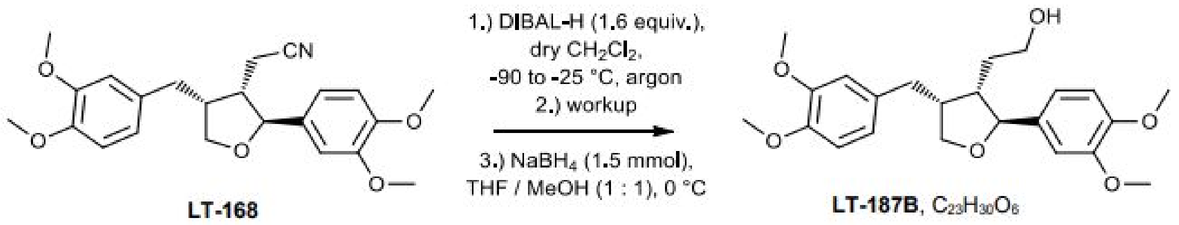

Figure S7. Reduction in two steps to the C1-elongated analogue LT-187B.

Procedure: a reaction vessel was charged with a stirring bar, starting material **LT-168** (55.5 mg, 0.140 mmol, 1.00 equiv.) and evacuated and back-filled with argon using standard Schlenk technique. Then, dry CH_2Cl_2 (3.5 mL) was added *via* syringe and the mixture was cooled to -85 °C in a MeOH / liquid N_2 bath. A solution of DIBAL-H (1 M in heptane, 0.36 ml, 0.36 mmol, 2.60 equiv.) was added dropwise *via* syringe and the mixture allowed to warm

to -50 °C over 90 min. This was followed by the dropwise addition of aqueous NaOH (2 M, 0.42 mL, 6.0 equiv.) and stirring for another 3 min. The cooling bath was removed and upon warming, water (3 mL) and CH_2Cl_2 (20 mL) was added, the layers were separated and the aqueous phase was re-extracted with CH_2Cl_2 (2 x 10 mL). The combined organic phases were dried with $MgSO_4$, filtered into a new reaction vessel, and the solvent was evaporated to obtain a residue of crude aldehyde intermediate (54.5 mg).

To this residue was added a stirring bar, MeOH (2.0 mL) and THF (1.5 mL), and the vessel closed without further provisions for inert conditions. After cooling to 0 °C in an ice bath, $NaBH_4$ (7.9 mg, 0.209 mmol, 1.50 equiv.) was added in one go and the mixture was stirred at 0 °C for 50 min.

Saturated aqueous NH_4Cl (0.5 mL) was then added slowly, the cooling bath was removed and stirring was continued for another 5 min. CH_2Cl_2 (25 mL) and brine (2 mL) was added, the layers were separated and the aqueous phase was re-extracted with CH_2Cl_2 (2 x 10 ml). The combined organic phases were dried with Na_2SO_4, filtered and the solvent was evaporated. Purification by flash column chromatography (9 g silica, flow rate 20 mL / min, EtOAc / LP, 40 : 60 to 100 : 0 in 30 min) afforded the title compound **LT-187B**.

**Yield**:	20.5 mg, 36 %
**Appearance**:	colorless oil
$\boldsymbol{R_f}$ **(silica)**:	0.40 (EtOAc)

$[\alpha]_D^{25}$: +39.5 (c 0.90, *i*-PrOH)

LC-HRMS (ESI): calculated for M+Na$^+$: 425.1940, found: 425.1958, Δ: 4.21 ppm

(log *P*)$_{calc}$: 2.93 ± 0.42

^{1}H NMR (200 MHz, $CDCl_3$): δ 1.36 (bs, 1H, OH), 1.66 (dq, ^2J = 13.4 Hz, ^3J = 6.6 Hz, 1H, C3-CH), 1.87 (dq, ^2J = 14.0 Hz, ^3J = 6.8 Hz, 1H, C3-CH), 2.24 – 2.42 (m, 1H, H3), 2.46 (dd, ^2J = 12.5 Hz, ^3J = 11.4 Hz, 1H, C4-CH), 2.47 – 2.71 (m, 1H, H4), 2.88 (dd, ^2J = 12.6 Hz, ^3J = 3.2 Hz, 1H, C4-CH), 3.67 (t, ^3J = 6.7 Hz, 2H, C3-$CH_2\underline{CH_2}$), 3.80 (dd, ^2J = 8.6 Hz, ^3J = 3.4 Hz, 1H, H5), 3.86 (s, 3H, Ar-OCH_3), 3.87 (s, 6H, Ar-OCH_3), 3.89 (s, 3H, Ar-OCH_3), 4.01 (dd, ^2J = 8.6 Hz, ^3J = 5.4 Hz, 1H, H5), 4.62 (d, ^3J = 8.3 Hz, 1H, H2), 6.67 – 6.92 (m, 6H, Ar-H).

^{13}C NMR (50 MHz, $CDCl_3$): δ 29.7 (t, C3-$\underline{C}$), 33.1 (t, C4-$\underline{C}$), 43.3 (d, C4), 47.5 (d, C3), 55.9 (q, 4 x Ar-OCH_3), 61.5 (t, C3-CH_2-$\underline{C}$), 72.3 (t, C5), 84.8 (d, C2), 109.3 (d, C2'), 110.9 (d, C5'), 111.3 (d, C5"*), 112.2 (d, C2"*), 118.7 (d, C6'), 120.8 (d, C6"), 133.0 (s, C1"), 135.0 (s, C1'), 147.4 (s, C4"), 148.6 (s, C4'), 148.9 (s, C3"), 149.1 (s, C3').

(*Z*)-2-((2*S*,3*S*,4*R*)-4-(3,4-Dimethoxybenzyl)-2-(3,4-dimethoxyphenyl)tetrahydrofuran-3-yl)ethyl 2-methylbut-2-enoate (**LT-188A** (**1**)):

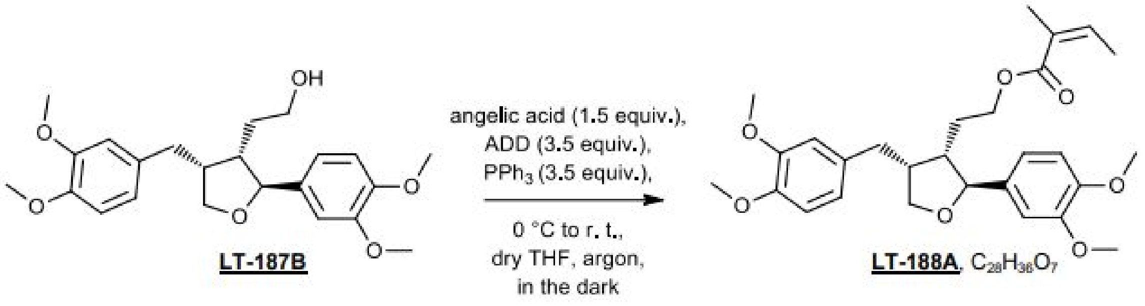

Figure S8. Mitsunobu procedure to obtain the C1 homolog of Leoligin, LT-188A (**1**).

Procedure: a reaction vessel was charged with a stirring bar, starting material **LT-187B** (17.5 mg, 0.043 mmol, 1.00 equiv.), angelic acid (6.5 mg, 0.065 mmol, 1.50 equiv.) and PPh_3 (39.8 mg, 0.152 mmol, 3.50 equiv.), and then evacuated and back-filled with argon using standard Schlenk technique. Dry THF (0.75 mL) was then added and the solution cooled to 0 °C in an ice bath. To the stirred mixture was then added a solution of ADD (38.3 mg, 0.152 mmol, 3.50 equiv.) in dry THF (1.0 mL) *via* syringe over approximately 1 min, and the reaction stirred for 23 h while being kept away from light and allowed to warm slowly to room temperature. Et_2O (5 mL) was then added to the reaction content, which was then filtered and rinsed with more Et_2O (15 mL). The solvents were evaporated and flash column chromatography was performed (9 g silica, flow rate 20 mL / min, EtOAc / heptane, 10 : 90 to 40 : 60 in 30 min), followed by preparative HPLC (flow rate 21.2 mL / min, MeOH / water, 63 : 37 to 70 : 30 in 60 min) to afford the title compound **LT-188A** (**1**).

Yield: 12.0 mg, 47 %

Appearance: colorless oil

R_f (silica): 0.47 (EtOAc)

$[\alpha]_D^{20}$: +49.9 (c 0.31, MeOH)

LC-HRMS (ESI): calculated for $M+Na^+$: 507.2359, found: 507.2365, Δ: 1.25 ppm

$(\log P)_{calc}$: 5.52 ± 0.47

GC-MS (EI, 70 eV, Method J): 46.61 min; 484.2 (M^+, 20), 205.1 (100), 165.1 (14), 151.0 (58).

^{1}H NMR (400 MHz, $CDCl_3$): δ 1.78 (dq, ^2J = 13.8 Hz, ^3J = 6.9 Hz, 1H, C3-CH), 1.83 – 1.86 (m, 3H, H5'''), 1.93 (dq, ^3J = 7.2 Hz, ^5J = 1.5 Hz, 3H, H4'''), 1.99 (dq, ^2J = 13.8 Hz, ^3J = 6.9 Hz, 1H, C3-CH), 2.25 – 2.35 (m, 1H, H3), 2.49 (dd, ^2J = 13.2 Hz, ^3J = 11.7 Hz, 1H, C4-CH), 2.59 – 2.69 (m, 1H, H4), 2.89 (dd, ^2J = 13.4 Hz, ^3J = 4.2 Hz, 1H, C4-CH), 3.80 (dd, ^2J = 8.7 Hz, ^3J = 3.9 Hz, 1H, H5), 3.87 (s, 3H, Ar-OCH_3), 3.87 (s, 6H, Ar-OCH_3), 3.88 (s, 3H, Ar-OCH_3), 4.03 (dd, ^2J = 8.7 Hz, ^3J = 5.8 Hz, 1H, H5), 4.19 (dt, ^2J = 11.1 Hz, ^3J = 6.9 Hz, 1H, C3-CH_2-C<u>H</u>), 4.23 (dt, ^2J = 11.1 Hz, ^3J = 6.9 Hz, 1H, C3-CH_2-C<u>H</u>), 4.65 (d, ^3J = 8.0 Hz, 1H, H2), 6.05 (qq, ^3J = 7.2 Hz, ^4J = 1.4 Hz, 1H, H3'''), 6.71 (d, ^4J = 1.9 Hz, 1H, H2''), 6.73 (dd, ^3J = 8.1 Hz, ^4J = 1.9 Hz, 1H, H6''), 6.81 (d, ^3J = 8.1 Hz, 1H, H5''), 6.81 – 6.88 (m, 3H, H2', H5', H6').

^{13}C NMR (100 MHz, $CDCl_3$): δ 15.9 (q, C4'''), 20.7 (q, C5'''), 26.0 (t, C3-<u>C</u>), 33.3 (t, C4-<u>C</u>), 43.0 (d, C4), 47.9 (d, C3), 56.0 (q, Ar-OCH_3), 56.09 (q, Ar-OCH_3), 56.11 (q, Ar-OCH_3),

56.14 (q, Ar-OCH_3), 62.8 (t, C3-CH_2-C), 72.4 (t, C5), 84.8 (d, C2), 109.4 (d, C2'), 111.1 (d, C5'), 111.5 (d, C5''*), 112.3 (d, C2''*), 118.8 (d, C6'), 120.9 (d, C6''), 127.9 (s, C2'''), 132.9 (s, C1''), 135.0 (s, C1'), 138.2 (d, C3'''), 147.6 (s, C4''), 148.8 (s, C4'), 149.1 (s, C3''), 149.3 (s, C3'), 168.1 (s, C1''').

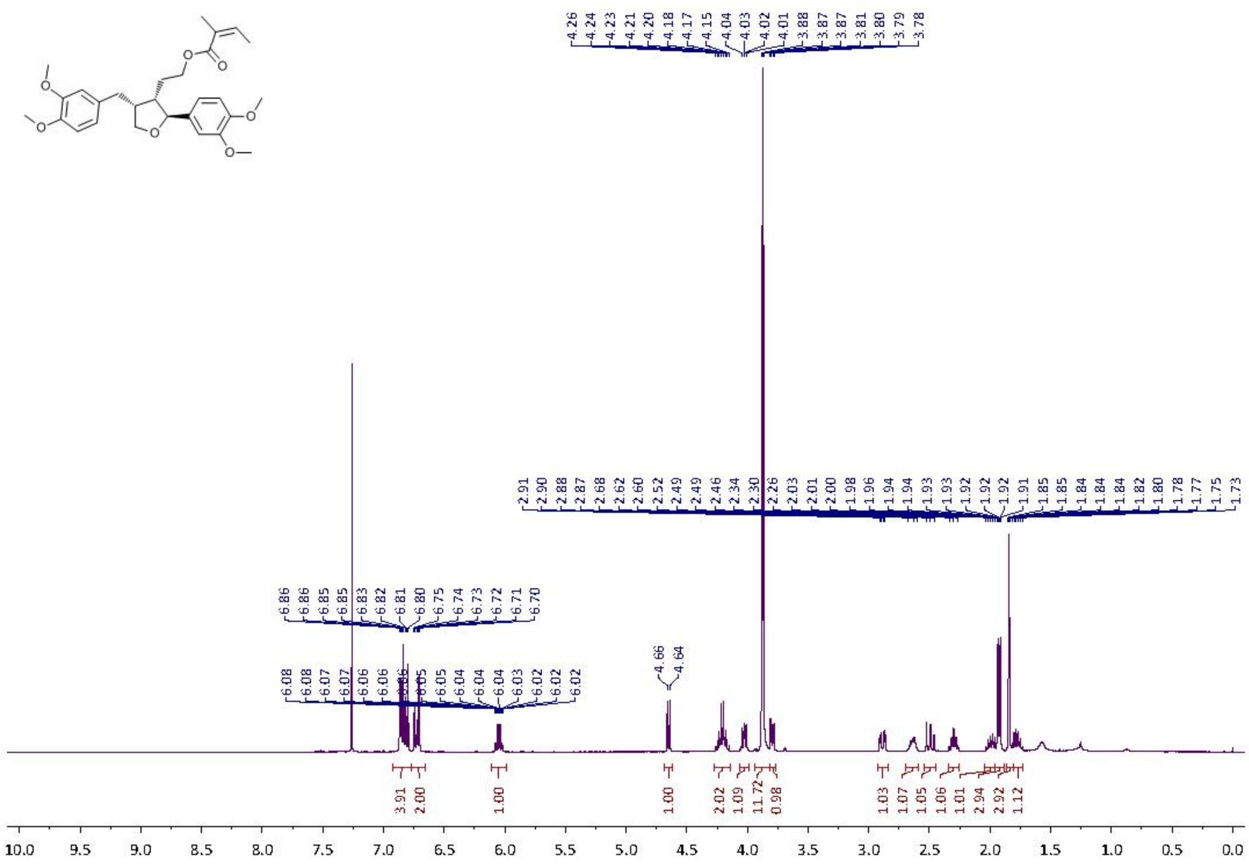

Figure S9. ^{1}H-NMR spectra of LT-188A (**1**).

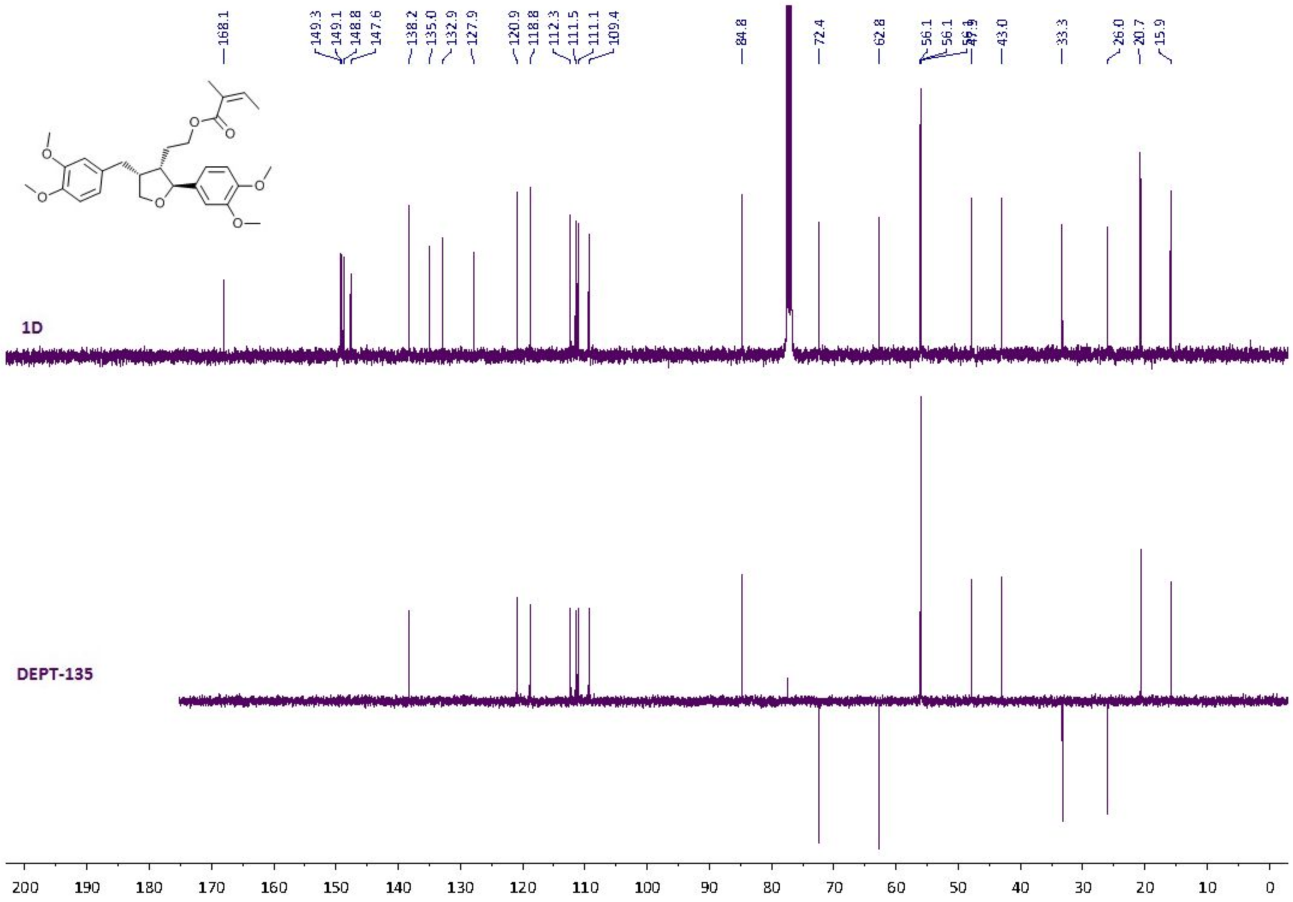

Figure S10. ^{13}C-NMR spectra of LT-188A (**1**).

Supplementary References

1. Kawamata Y, Fujii R, Hosoya M, Harada M, Yoshida H, Miwa M, et al. A G protein-coupled receptor responsive to bile acids. J Biol Chem. 2003;278(11):9435-40.

2. Genet C, Strehle A, Schmidt C, Boudjelal G, Lobstein A, Schoonjans K, et al. Structure-activity relationship study of betulinic acid, a novel and selective TGR5 agonist, and its synthetic derivatives: potential impact in diabetes. J Med Chem. 2010;53(1):178-90.
